# An integrated *in vitro* platform and biophysical modeling approach for studying synaptic transmission in isolated neuronal pairs

**DOI:** 10.1016/j.isci.2026.115488

**Published:** 2026-04-01

**Authors:** Giulia Amos, Vaiva Vasiliauskaitė, Jens Duru, Maria Leonor Azevedo Saramago, Tim Schmid, Alexandre Suter, Ferran Cid Torren, Joël Küchler, Tobias Ruff, János Vörös, Katarina Vulić

**Affiliations:** 1 Laboratory of Biosensors and Bioelectronics (LBB), Institute for Biomedical Engineering, D-ITET, ETH Zurich, 8092 Zurich, Switzerland

**Keywords:** Cellular physiology, Cellular neuroscience, Stem cells research

## Abstract

Studying synaptic transmission is facilitated in experimental systems that isolate individual neuronal connections. We developed an integrated platform combining polydimethylsiloxane (PDMS) microstructures with high-density microelectrode arrays to isolate, record, and manipulate neuronal pairs from human induced pluripotent stem cell (hiPSC)-derived neurons. The system maintained hundreds of parallel neuronal pairs for over 100 days, demonstrating functional synapses through pharmacological validation. We coupled this platform with a biophysical Hodgkin-Huxley model and simulation-based inference to extract mechanistic parameters from the electrophysiological data. As a proof-of-concept application, we analyzed shifts in model parameter distributions following a stimulation protocol. The biophysical model revealed α-amino-3-hydroxy-5-methyl-4-isoxazole propionic acid (AMPA) and N-methyl-D-aspartate (NMDA) receptor-specific alterations after stimulation, providing quantitative insights into synaptic plasticity mechanisms. This integrated approach combines isolated hiPSC-derived synaptic pairs, stable parallel long-term recordings, and mechanistic modeling to enable systematic studies of human synaptic transmission.

## Highlights

• PDMS microstructures isolate hundreds of hiPSC neuronal pairs on a single chip
• Neuronal pairs in isolation remain viable and electrically active for over 120 days
• *In vitro*-*in silico* framework extracts synaptic parameters from extracellular activity
• Hodgkin-Huxley modeling links 5 Hz stimulation to AMPA-driven synaptic potentiation

## Graphical abstract

**Figure:**
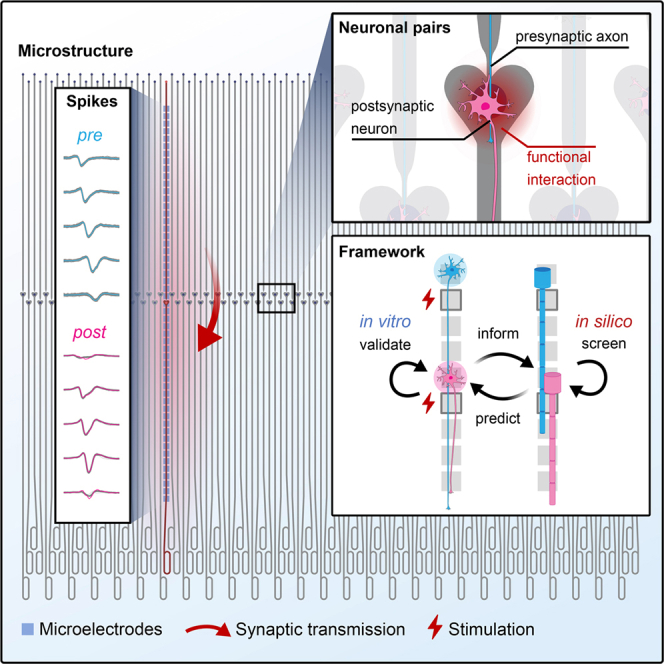

## Introduction

The brain’s ability to learn, adapt, and perform complex functions arises from the dynamic interplay of neuronal networks that selectively transmit and store information. Learning occurs when experience induces lasting changes in neuronal activity and behavior. At the cellular level, these adaptive changes are mediated primarily by synaptic plasticity,^1^^,^^2^ defined as the ability of synapses to strengthen or weaken over time in response to patterns of activity. Understanding how plasticity shapes the connectivity and function of neuronal networks is central to understanding the mechanisms underlying learning, memory, and brain development. Research in this area follows two complementary avenues: experimental approaches and computational modeling.

Traditional experimental approaches to studying synaptic function and plasticity span a spectrum from complex *in vivo* and *ex vivo* preparations to simplified and controlled *in vitro* systems. *In vivo* approaches have revealed fundamental principles such as long-term potentiation (LTP) and long-term depression (LTD),^3^^,^^4^ and have mapped the connectivity and plasticity rules of various brain regions.^5^^,^^6^^,^^7^^,^^8^ *Ex vivo* brain slices preserve local circuit architecture and have enabled detailed characterization of synaptic responses and plastic changes following stimulation protocols.^9^^,^^10^^,^^11^^,^^12^ However, these experimental models present two significant limitations: first, they rely on animal preparations rather than human tissue, potentially limiting translational relevance; and second, they cannot effectively isolate the contributions of individual synapses or neuronal pairs due to extensive background connectivity and overlapping activity from surrounding neuronal networks. Consequently, unraveling the precise cellular and molecular mechanisms underlying synaptic plasticity remains challenging in these complex preparations.

*In vitro* experiments offer compelling solutions to these challenges by providing greater experimental control and human relevance. First, they enable the use of human-derived cells through induced pluripotent stem cell (iPSC)-derived neurons, eliminating species-specific differences that may limit translational applicability. Second, they offer simplified systems with substantially fewer confounding variables than intact brain preparations.^13^^,^^14^^,^^15^^,^^16^ However, even random *in vitro* cultures, despite containing orders of magnitude fewer neurons than the brain, can develop complex network architectures that obscure fundamental mechanisms. Therefore, imposing structural organization and directional connectivity in cultured systems can be essential for mechanistic studies. One promising approach involves guiding neuronal growth and controlling connectivity by confining cells within polydimethylsiloxane (PDMS) microstructures. Substantial progress has been made in this direction, ranging from larger compartmentalized systems that isolate neuronal clusters^17^^,^^18^^,^^19^ to progressively smaller configurations designed to isolate individual neurons^20^ and axons^21^^,^^22^^,^^23^ or even synaptic locations.^24^^,^^25^ Many of these PDMS-based systems are compatible with extracellular microelectrode arrays (MEAs).^26^^,^^27^ While patch clamp electrophysiology has been the gold standard for obtaining precise information about ionic channel properties, synaptic currents, and membrane dynamics, it is inherently time-consuming, low-throughput, and incompatible with long-term monitoring. MEAs and other higher-throughput recording methods enable the generation of statistically robust datasets and recordings over multiple weeks *in vitro*, such as during maturation.^13^^,^^28^

While experimental approaches provide empirical data on synaptic function,^15^^,^^16^ computational models complement these efforts by allowing researchers to manipulate parameters and test hypotheses that would be challenging to address experimentally.^29^^,^^30^ Computational models have elucidated the rules governing spike-timing-dependent plasticity,^31^^,^^32^^,^^33^ modeled the impact of synaptic noise and variability,^34^ and predicted how changes at individual synapses can scale up to affect network dynamics and information processing.^35^^,^^36^ These types of models play an important role in bridging the gap between molecular mechanisms and emergent network behavior. However, despite their power, computational models are inherently limited by the need to simplify biological detail for tractability,^30^^,^^37^^,^^38^ and their predictions often lack direct experimental validation due to difficulties in finding precise biological equivalents of the computational networks. This gap in the experimental validation highlights the need for experimental systems that can serve as a bridge between computational models and biological approaches.

Small and well-characterized *in vitro* experimental systems represent ideal candidates for biophysical modeling approaches.^39^^,^^40^ Networks with reduced complexity allow for a comprehensive parameterization of synaptic properties without the confounding influences present in more complex systems. Fewer variables, improved experimental control, and clearer correspondence between model components and biological substrates facilitate direct comparison between experimental observations and model predictions. Consequently, well-characterized small systems provide platforms for iterative model refinement, where discrepancies between predicted and observed behavior can be systematically addressed by gradually extending the model with missing details. Model predictions can then guide experimental design, establishing a bidirectional feedback loop between theory and experiment.

However, no existing platform is specifically designed to study synaptic transmission with this bidirectional experimental-computational framework. While systems that isolate single cells exist, including iPSC-derived neurons on microislands,^15^^,^^16^ autaptic cultures,^41^^,^^42^ and combined HD-MEA with patch-clamp approaches,^43^ these provide limited throughput and lack architectures that enable controlled formation of unidirectional neuronal pairs. Conversely, larger network preparations offer higher throughput but introduce network complexity that complicates biophysical modeling and the extraction of synaptic parameters.^30^^,^^44^^,^^45^ This gap has limited the ability to characterize synaptic transmission using MEA recordings^46^ and to effectively integrate experimental observations with computational models.

In this study, we present a platform that integrates experimental and computational models with compartmentalized PDMS microstructures to study unidirectional synaptic transmission. By employing PDMS microstructures with precisely defined dimensional constraints, we enable stochastic isolation of hundreds of individual neuronal pairs on a single device. Compatibility with high-density MEAs provides parallel recording capabilities that yield statistically robust datasets while preserving access to individual neuronal dynamics over several months. Using data from this platform, we developed a biophysical model based on Hodgkin-Huxley (HH) formalism^40^^,^^47^ to link experimental observations in our simplified platform with mechanistic modeling. As a proof-of-concept application, we use this model to quantitatively characterize synaptic transmission properties during maturation and after perturbation, extracting biophysically meaningful parameters from experimental data using simulation-based inference (SBI) techniques.^48^^,^^49^ Together, these results establish this platform as a tool for investigating fundamental questions about human synaptic function and plasticity.

## Results

### Single neuronal pairs can be isolated using microstructures with 10 *μ*m openings

**Figure 1:**
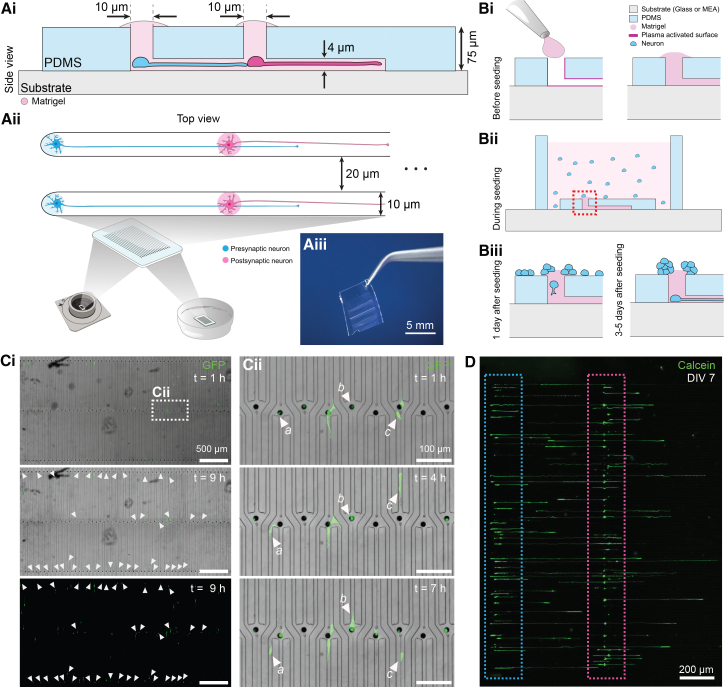
Single-cell PDMS microstructure for neuronal isolation and migration (A) Device design concept and adaptability to substrates. Ai) The PDMS microstructure features 10 *μ*m wide and 75 *μ*m high wells that narrow down to 4 *μ*m high microchannels. Microstructures are placed on a substrate and filled with Matrigel. Aii) The microchannel design connects two wells intended for presynaptic (blue) and postsynaptic (pink) neurons. Microchannels isolating separate pairs are min. 20 *μ*m apart. Aiii) Photograph showing an actual fabricated PDMS device as used in our experiments. Scale bars, 5 mm. (B) The cell seeding and migration process. Bi) Prior to cell seeding, the microchannels are filled with Matrigel to create a supportive matrix for cell growth. Bii) During seeding, neurons randomly position themselves on top of the microstructure. 0.5 mm high PDMS seeding frame is placed around the microstructure to restrict the area where the cells can land. Biii) Following seeding, neurons gradually migrate into the microchannels, with movement observed after one day and continued migration within the first week. (C) Timelapse of neuronal migration upon seeding. Ci) Phase contrast images reveal the progressive migration of cells into the microwells during the first hour (t = 1 h) and after 9 h (t = 9 h) with GFP-labeled neurons. Neurons that enter the microchannels between the first and ninth hour are indicated with white arrows. Scale bars, 500 *μ*m. Cii) Higher magnification views of the boxed region allow for tracking individual cells (three exemplary cells marked by letters) as they navigate through the microchannels. Scale bars, 100 μm. (D) Calcein-stained neurons on DIV 7 grew along a presynaptic (indicated with a blue rectangle) and postsynaptic (indicated with a pink rectangle) region. Scale bars, 200 *μ*m.

In this work, we introduce a platform for isolating minimal neuronal networks, down to single neuronal pairs, to examine their morphological and electrophysiological characteristics. We achieve this precise isolation by reducing the diameter of the PDMS well to just 10 *μ*m (Figure 1Ai), a significant modification over previous designs^22^^,^^26^^,^^50^^,^^51^ that comprised well diameters ranging from 150 to 400 *μ*m. The approach to neuronal isolation shown in this work presents unique challenges, primarily because the underlying isolation mechanism differs fundamentally from established methods. Unlike systems where cells settle randomly in wells when seeded in suspension,^26^^,^^52^ or where spheroids can be manually placed using a pipette,^22^^,^^51^ the system relies on cells that migrate stochastically into wells, predominantly during the initial week in culture.

The efficacy of this microfluidic system depends on two critical factors: ensuring neuronal viability within the microchannels and achieving isolated neuronal pairs that remain functionally independent from surrounding neurons. The migration and survival of neurons into the microchannels required an appropriate matrix. While previous approaches^22^^,^^26^^,^^50^^,^^52^ relied on PDL and/or laminin coating to support cell growth, with these we observed low cell numbers in 10 *μ*m wells and poor viability of isolated cells (Figures S1A and S1B). Higher abundance was achieved when microchannels were filled with Matrigel (Figure 1Bi). Since Matrigel was used at a concentration of 3 mg/mL, which is highly viscous, we optimized the hydrophilicity of the microchannel surfaces to effectively fill the microstructures. This was accomplished by exposing the substrate and the bottom of the PDMS microstructure (i.e., the microchannels) to oxygen plasma prior to mounting the PDMS to the substrate, followed by immediate filling of the channels with diluted, uncrosslinked Matrigel. Cells that landed on the top PDMS surface coated with Matrigel residuals (Figure 1Bii) gradually migrated downward into the wells (Figure 1Biii). This process is documented in the Video S1, which captures a time-lapse sequence between DIV 0 and DIV 1, immediately after seeding. Time-lapse imaging revealed active soma translocation, with cell bodies physically moving into wells and navigating along microchannels (Figure 1Ci-Cii), likely seeking contact with other neurons as NGN2 neurons tend to cluster. While some neurons established positions in the wells and began extending neurites, others actively navigated along the microchannels (Figure 1Cii). This soma translocation occurred predominantly within the first 1–2 days after seeding. After approximately one week *in vitro*, most microchannels were filled with cells (Figure 1D), resulting in tens to over a hundred potential synaptic pairs in parallel (45% of microchannels contained fewer than two cells, 37% contained exactly two, and 18% contained more than two on DIV 7; *N* = 322, see Figure S3).

Video S1. Time-lapse of neuronal migration into microchannels Neurons are shown migrating into the PDMS microchannels between DIV 0 and DIV 1 following seeding. Cell bodies can be observed translocating into the wells and navigating along the microchannels, related to Figure 1.

The irreversible bonding of PDMS microstructures assured the isolation of neurons and prevented neurons from growing in the neighboring microchannels. Plasma treatment following Matrigel channel filling selectively degraded the coating on the top PDMS surface, as the plasma cannot effectively penetrate the matrigel-filled microstructures. The resulting suboptimal extracellular matrix conditions on top of the PDMS surface induce aggregation and eventual detachment of the remaining cells (Figure 1Biii), thereby preventing unwanted cross-connectivity between the otherwise isolated channels. The comparison of the top of the PDMS microstructure with and without plasma treatment post-matrigel coating is shown in Figure S2. The combination of optimized matrix conditions and selective surface treatment provides a methodology for isolating neuronal pairs that remain viable and functionally independent from surrounding neurons.

### Structural motifs enforce axonal directionality in pre- and postsynaptic neuronal pairs

We aimed to further optimize the system by introducing directionality, thereby ensuring well-defined pre- and postsynaptic neurons and their locations. This directional control is important for creating targeted networks or co-cultures with specific neuronal populations, such as distinct subtypes of cortical neurons or strategic placement of disease-model neurons at predetermined locations within the network.

**Figure 2:**
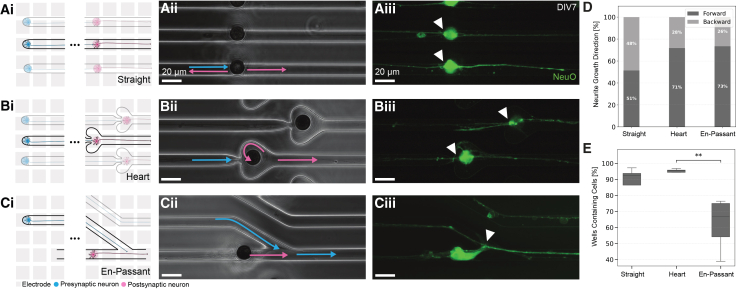
Microstructure designs for directional neuronal growth (A) The straight microstructure design provides a baseline without enforced directionality. Ai) Schematic representation shows a simple channel connecting presynaptic (blue) and post-synaptic (pink) neuronal compartments. Aii) Phase contrast image reveals the fabricated straight microstructure design. Aiii) Fluorescence image demonstrates NeuO-labeled neurons extending processes through the channel, with cell-to-cell contacts visible (white arrowhead). (B) The heart-shaped microstructure incorporates axon guidance principles. Bi) Schematic illustration depicts the heart-shaped feature designed to prevent backward axonal growth. Bii) Phase contrast image shows the fabricated heart-shaped microstructure with guidance features for the postsynaptic neurons highlighted (pink arrow). Biii) Fluorescence imaging confirms neuronal growth through the structure with presynaptic neurites extending to the postsynaptic neuron (white arrowheads). (C) The en-passant microstructure design facilitates axon-dendritic connections. Ci) Schematic representation shows the angled channel design for axon-dendritic en-passant synapses. Cii) Phase contrast micrograph demonstrates the fabricated en-passant structure with highlighted directional cues. Blue and pink arrows indicate desired growth directions for pre-, respectively, postsynaptic neurons. Ciii) Fluorescence imaging confirms neuronal growth through the angled channels with the postsynaptic cell body in contact with the presynaptic neurites (white arrowhead). (D) Quantification of neurite growth direction across the three designs shows: straight (51% forward, 49% backward, *N* = 175), heart (72% forward, 28% backward, *N* = 245), and en-passant (73% forward, 27% backward, *N* = 94). (E) Quantification of channel occupancy (percentage of wells with cells) reveals a significant difference (∗*p* < 0.05) between designs, with en-passant showing lower occupancy compared to heart and straight designs. Statistical significance was assessed using a one-way ANOVA followed by pairwise t-tests with Bonferroni correction for multiple comparisons. For more details, check Table S1. Data are represented as mean ±95% confidence interval. Scale bars, 20 *μ*m.

We designed and tested three main microstructure configurations to establish pre- and postsynaptic neuronal connections (Figure 2). All proposed designs are compatible with high-density complementary metal-oxide semiconductor (HD-CMOS) MEAs to record neuronal signals with high spatial and temporal resolution (Figures 2Ai, 2Bi, and 2Ci). Since the electrode size (12.5 *μ*m) is larger than the microchannel width (10 *μ*m) in all designs, each electrode ideally records from only one microchannel, therefore narrowing down the variability of neuronal signals it collects.

The first design (Figure 2A) featured a straight microstructure with no enforced directionality other than the well locations. The second design (Figure 2B) incorporated a structure based on axon guidance principles, which states that axons tend to follow edges, but will not follow the edge around sharp corners.^18^^,^^53^^,^^54^^,^^55^ Due to space constraints, we implemented a minimal design where a heart structure with a narrow entering region reduces the probability of the postsynaptic axons to grow back into the presynaptic channel (indicated by pink arrows in Figure 2Bii). The third design was inspired by the axo-dendritic “en-passant” synapses described previously,^56^ where edge guidance and differences in axonal and dendritic lengths create unidirectional connections from pre-to postsynaptic neurons. In this design, directionality of both the pre- and postsynaptic neurons is controlled by introducing a sharp turn in the junction of the channels (indicated by pink and blue arrows in Figure 2Biii). The desirable contact points between the presynaptic axon and the postsynaptic cell are indicated by white arrows in Figure 2Ci-iii for each design.

We quantified neurite growth direction using a combination of Fiji and manual annotation to assess the success of the three designs in enforcing directionality in the formation of synapses (Figure 2D). The straight design exhibited no preferential directionality, with nearly equal forward (51%) and backward (49%) growth (*N* = 175). The close to 50:50 bidirectional growth pattern confirms that without structural guidance, neuronal processes extend stochastically and equally in both directions. In contrast, both the heart design (*N* = 245) and en-passant design (*N* = 94) demonstrated significant directional preference, with comparable 72% and 73% forward growth. While robust, the directionality is lower than the directionalities above 90% reported in previous studies.^18^^,^^56^^,^^57^ This difference might be attributed to our simplified designs, which prioritized spatial efficiency and ease of fabrication.

Although the percentage of channels containing cells was similar across all designs, we observed a significant increase in cell occupancy within the heart and straight designs compared to the en-passant configuration (Figure 2E). We hypothesize that this difference results from subtle variations in design. The straight and heart designs feature two rows of wells, while the en-passant configuration consists of three rows (see Figures S4–S6). Since the wells are more distributed, the probability of a cell that landed on the top surface migrating to a well is lower.

Despite these minor differences, the comparable channel occupancy across all designs indicates that our structural modifications did not significantly affect neuronal viability or the accessibility of the channels. We present a wide spectrum of channel designs, from standard microchannels down to nanochannels, which enable precise control over axodendritic synaptic connections, in Figures S4, S5, S6, S7, and S8. These designs support diverse experimental paradigms from basic synaptic morphology studies in monocultures to complex co- or tri-culture investigations.

### Neurons in isolated microchannels are active for over 4 months in culture, allowing detailed characterization

Neurons typically develop and function within complex networks, rarely surviving in isolation under standard culture conditions due to their dependence on trophic support and network activity.^27^^,^^58^ Therefore, a critical concern for the reductionist network approach was whether these cells would remain viable and functionally active over extended periods despite the drastically reduced network complexity.

**Figure 3:**
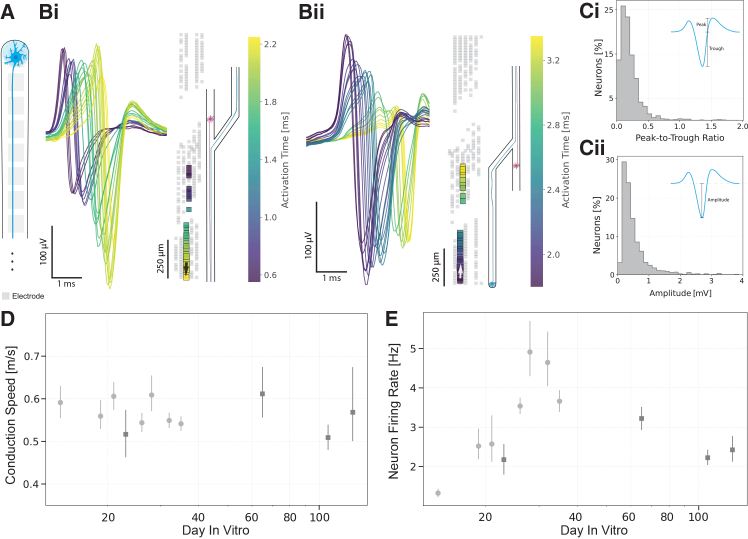
Electrophysiological characterization of neurons in isolated microchannels (A) Schematic of a single neuron in a microchannel. (B) Activity of selected neurons in microchannels. Bi) Representative spike waveforms from a single neuron recorded across 25 electrodes along one microchannel. Templates from twenty-five electrodes belonging to the same channel are plotted in time. Waveforms are color-coded by their activation time (temporal sequence), with earlier activations in purple/blue transitioning to later activations in yellow, illustrating the spatial-temporal progression of the action potential as it travels along the axon (black arrow indicates the direction of propagation). The electrode arrangement schematic (left) shows the spatial layout along the microchannel. Bii) Example from a different neuron on the same chip with a different template progression. White arrow indicates the direction of action potential propagation. (C) Example metrics extracted per unit/neuron at DIV 65 (*N* = 1432), showing distributions of Ci) peak-to-trough ratios and Cii) amplitudes. (D) Evolution of conduction speed with culture age. One set of chips (*N* = 4) is used to assess the behavior during the first five weeks (light gray circles) and another over a longer time in culture (up to 129 days, dark gray squares). (E) Evolution of the mean neuron firing rate of the neurons in D) over 129 days. Data in D) and E) are represented as mean ±95% confidence interval. Histograms in Ci-ii show the distribution of values as a percentage of total units, with an overlaid kernel density estimate (KDE) curve.

We showed that neurons constrained to minimal networks not only survive but maintain robust electrical activity for several months. Placing PDMS microstructures on top of HD-MEAs enabled comprehensive electrophysiological characterization of the cultures. The physical confinement of neuronal pairs within microchannels ensured that we were consistently recording signals from the same neurons throughout the experiment. The spike sorting approach we employed automatically detected and assigned extracellularly recorded action potentials to individual neurons (Figure 3Bi and Bii). Waveform shapes varied from one neuron to another but also changed along the microchannel for the same neuron, which could be attributed to recording from different cellular compartments (from dendrites, through somas to axons),^28^ varying positions of the recorded cell parts with respect to the electrodes, or the relative position of the electrodes with respect to the PDMS microstructure.^59^ Figure 3Ci and 3Cii illustrate representative waveform metrics extracted from our recordings. Additional extracted waveform metrics are available in Figure S9. Notably, the majority of recorded action potential amplitudes exceeded 100 *μ*V, whereas these values are only achievable over multiple averages in random cultures,^28^ highlighting the significant advantage of our PDMS microstructures in improving the signal-to-noise ratio.

Neurons exhibited sufficient activity for the reliable calculation of action potential propagation (Figure 3D) and firing rate (Figure 3E) from the second week (DIV 14) onward. We observed a relatively constant conduction speed between DIV 14 and DIV 129, which, given the established relationship between conduction velocity and unmyelinated axon diameter,^60^^,^^61^ indicated that axonal morphological properties and ion channel distribution along the axons were established early in development. In contrast, the firing rate showed a steady increase until DIV 28, reflecting ongoing development of network connectivity and neuronal excitability, likely through the formation and strengthening of synaptic connections. The firing rate then stabilized between 2 and 3 Hz and remained relatively constant throughout the culture’s lifespan. The minor periodic fluctuations observed in both conduction speed and firing rate over weeks *in vitro* likely resulted from environmental factors such as temperature fluctuations or changes in medium composition due to evaporation and cellular processes, rather than developmental changes in the neurons themselves.

These findings are consistent with previous reports on NGN2-induced neurons,^51^^,^^58^^,^^62^ some of which also describe conduction velocities around 0.5 m/s and spontaneous firing rate stabilization after four weeks in culture. Importantly, we demonstrated that neurons remained viable for over 120 days, which is longer than the durations reported in most studies using NGN2 neurons without additional support from glial co-culture or specialized media.^27^^,^^42^^,^^58^^,^^63^ This, and the stability of measurements, confirmed the effectiveness of PDMS isolation for long-term neuronal recordings.

### Isolated neuronal pairs form functional synaptic connections

Having demonstrated that neurons maintain robust electrical activity for extended periods within the microstructures (Figure 3), we next investigated whether these isolated neuronal pairs in microchannels establish functional synaptic connections. Previous computational, *ex vivo,* and *in vitro* work has demonstrated that multiple synapses and simultaneous incoming stimuli are typically required to elicit action potentials in postsynaptic neurons.^30^^,^^43^^,^^64^^,^^65^ Therefore, it was crucial to verify both the physical presence of synapses and their functional capacity to influence postsynaptic firing in the isolated two-neuron system.

We identified putative synaptic pairs by testing whether the past activity of a presynaptic neuron significantly reduces uncertainty about the current state of a postsynaptic neuron, beyond what can be inferred from its own past. This was quantified using transfer entropy—a model-free measure of directed information flow between time series^66^^,^^67^ (see methods). This approach has already been applied to the large-scale neuronal data.^68^^,^^69^

**Figure 4:**
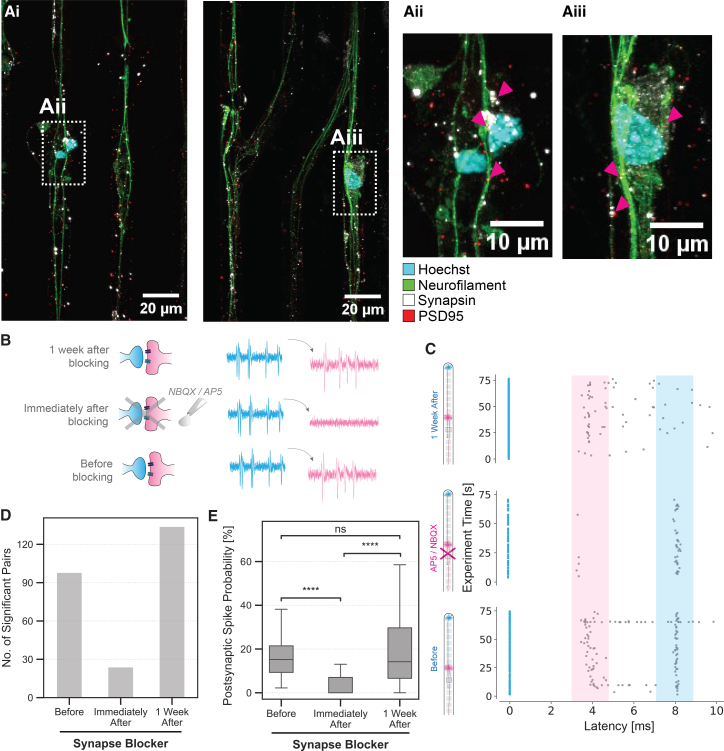
Validation of synaptic connectivity of neuronal pairs in isolated microchannels (A) Immunocytochemical evidence of synaptic connections. (i) Fluorescent microscopy images show the colocalization of synaptic markers. Scale bars, 20 *μ*m. (ii) A higher magnification inset provides detailed the visualization of the synaptic structures. Scale bars, 10 μm. (B) Schematic of the experiment. NBQX and AP5 were added as NMDA and AMPA receptor antagonists on DIV 100, and the activity was recorded before the application of synaptic blockers, immediately after, and one week after washout. (C) Representative spontaneous spike time-triggered latency plot shows spikes before, in the presence of synaptic blockers, and one week (following washout) after the application of synaptic blockers, demonstrating the change in synaptic transmission properties. (D) Bar graph showing the total number of significant transfer entropy links before, after synaptic blocker application, and following washout for one chip. (E) Boxplots quantify the synaptic transmission probability across the three conditions, with statistical significance indicated (∗∗∗∗*p* < 0.0001, Kruskal-Wallis omnibus tests followed by Dunn’s tests, for more details check Table S2). Boxplots show median, interquartile range, and 1.5× IQR whiskers.

To validate the functional connections identified by transfer entropy, an artificial neural network was trained to forecast postsynaptic spike probabilities based on preceding pre- and postsynaptic activity. The results of this analysis further support the transfer entropy-based identification of synaptic pairs: for neuron pairs classified as coupled by transfer entropy, both traces significantly contributed to prediction accuracy (see Supplementary Material S17). While transfer entropy captures directed information flow, it does not by itself confirm causal synaptic transmission.^70^ We therefore complemented the functional analysis with immunocytochemistry, which revealed the colocalization of synapsin and PSD95 puncta—markers of pre- and postsynaptic proteins—across all microstructure designs, confirming the physical presence of synaptic contacts (Figure 4A).

To examine the functionality of putative synapses in the system, we performed a pharmacological intervention on DIV 100 using α-amino-3-hydroxy-5-methyl-4-isoxazole propionic acid (AMPA) and N-methyl-D-aspartate (NMDA) receptor antagonists (NBQX and AP5, respectively) to inhibit glutamatergic synaptic transmission (Figure 4B). We recorded and compared spontaneous neuronal activity at three time points: before antagonist application (baseline), immediately after antagonist application (acute 30 min exposure during recording), and one week following washout (to assess recovery). The antagonists were washed out after the immediate post-application recording by replacing the culture medium three times with fresh medium. Figure 4C shows a spontaneous-spike-time-triggered latency plot from a single electrode that captured spikes from both presynaptic and postsynaptic neurons within the same microchannel. The latency plot revealed two distinct activity bands at approximately 4 ms and 8 ms. The 4 ms band (highlighted in pink) represents the postsynaptic response, while the 8 ms band (highlighted in blue) likely represents recurrent presynaptic activity. Following antagonist application, the 4 ms band disappeared, confirming successful blockage of synaptic transmission, while the 8 ms band persisted with reduced density, reflecting decreased overall firing rate. The 4 ms coupling lag likely accounts for synaptic delay, postsynaptic integration time, and conduction.

We focused on two metrics to quantify these functional changes: the number of significant pre- and postsynaptic neuronal pairs, defined as those with transfer entropy significant at the 0.05 level, across the three time points; and the probability of postsynaptic spike occurrence following presynaptic spikes. The number of significant pairs shows a 4-fold decrease immediately upon antagonist addition and a 6-fold recovery one week after washout (Figure 4D). Similarly, the administration of synaptic blockers resulted in a significant reduction in the postsynaptic spike probability (Figure 4E) as well as a decrease in pairwise transfer entropy values (shown in Figure S10). Significant differences were observed between pre-blocker and blocked conditions as well as between blocked and washout conditions. Importantly, no significant difference was found between pre-blocker and one-week-after washout conditions, indicating complete recovery of synaptic function after blocker removal. The reversible disruption of postsynaptic spike probability and transfer entropy by synaptic blockers and subsequent recovery after washout demonstrate that neuronal pairs isolated in microchannels form functional synaptic connections, despite their minimal configuration. While we demonstrated functional connectivity, detailed characterization of synaptic properties such as release probability would require additional experimental protocols beyond the scope of this study.

**Figure 5:**
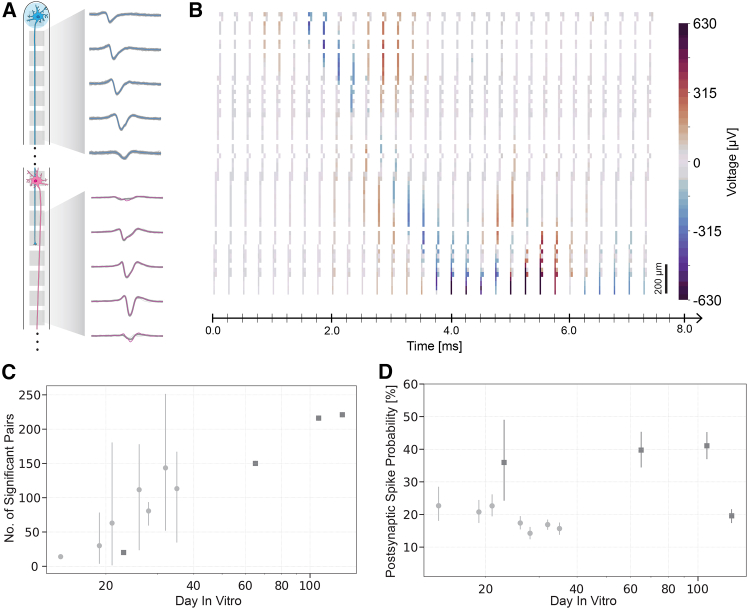
Synaptic connectivity dynamics in isolated neuronal pairs (A) An example of processed electrophysiological recordings from neurons in microchannels showing spike-sorted traces from both pre-synaptic (blue, top) and post-synaptic (pink, bottom) neurons, with the propagation of the traces along the channel by displaying 5 equally spaced electrodes for each neuron. (B) Video frames showing the temporal relationship between pre- and post-synaptic activity with a color-coded amplitude scale in microvolts (*μ*V). Scale bars, 200 *μ*m. See also Video S2. (C) Number of significant pairs found by transfer entropy analysis for one set of chips (*N* = 4) early in culture (first five weeks, light gray circles) and for another over a longer time in culture (up to 129 days, dark gray squares). (D) Postsynaptic spike probability of the neurons in (C) early in culture (first five weeks, light gray circles) and over a longer time in culture (up to 129 days, dark gray squares). Data in C) and D) are represented as mean ±95% confidence interval.

Putative synaptic transmission can also be confirmed by visual inspection on a case-by-case basis. Spike sorting of the recordings enabled the distinction of action potentials from neurons growing in close proximity (Figure 5A). Figure 5B illustrates spike propagation and putative synaptic transmission in a representative microchannel. The recordings demonstrate action potential propagation along the microchannel, with the initial spike traveling from the top electrode (negative deflection at 1.75 ms) and continuing down the channel (positive component at 2.75 ms). A secondary action potential emerges at 4.75 ms in the bottom half of the microchannel, exhibiting propagation and a 3 ms synaptic delay that indicates postsynaptic initiation rather than the passive propagation of the original signal.

Video S2. Visualization of putative synaptic transmission in a representative microchannel Color-coded voltage amplitude maps showing the temporal relationship between pre- and postsynaptic neuronal activity in a representative microchannel. The color scale indicates voltage amplitude in microvolts (*μ*V), illustrating action potential propagation and putative synaptic transmission between isolated neuronal pairs, related to Figure 4.

We systematically analyzed different synaptic metrics over time to quantify the long-term dynamics of functional connectivity. The number of functional pairs reveals a clear developmental trajectory during the first five weeks in culture (Figure 5C). While few significant connections are detected in the earliest recordings (DIV 15–20), the number increases substantially during the first month in culture. The number of detected pre- and postsynaptic pairs further increases after DIV 80, indicating the ongoing network formation and enhancement of functional connectivity throughout the culture period. One hypothesis is that putative remaining neurons on top of the PDMS surface continue to migrate and project axons inside the microchannels. Another hypothesis is related to the fact that individual NGN2 neurons can develop multiple axons even after 4–6 weeks in culture.^15^^,^^71^ Similarly, postsynaptic spike probability varies between 15 and 35% within the first 20 days, increases to 40% beyond DIV 60 as networks mature, and notably declines at DIV 129, potentially indicating culture senescence (Figure 5D).

This observation is particularly interesting when contrasted with the number of significant connections, which remains high at this time point, and the firing rate (Figure 3E), suggesting that while the quantity of neuronal pairs persists, the efficacy of their synaptic transmission decreases. This implication aligns with the literature that states that neurons suffer synaptic dysfunction and axonal connectivity loss prior to somatic cell death.^72^^,^^73^ Also interesting is the fact that postsynaptic spike probabilities observed in our system exceed those typically reported for single unitary connections in cortical preparations (<5–10%).^58^^,^^64^ This likely reflects several factors. Firstly, multiple synaptic contacts per neuronal pair, potentially enhanced by the ability of NGN2 neurons to develop multiple axons.^71^ Secondly, proximal synaptic placement due to spatial constraints, minimizing distance-dependent attenuation.^74^ Thirdly, selection bias in our transfer entropy-based pair identification, which preferentially detects stronger connections. Finally, there could potentially be different receptor properties in iPSC-derived neurons compared to mature cortical neurons. More detailed analysis of receptor distribution and synaptic ultrastructure would be required for a complete mechanistic understanding.

### Simulation-based inference finds the biophysical parameter space from the microelectrode-array data of neuronal pairs

We complemented the experimental platform with a biophysical HH model^75^ to further leverage the rich electrophysiological data from the experiments and investigate the mechanisms of synaptic transmission. The absence of complex neuronal networks in our *in vitro* system allowed for the use of a minimal computational model capable of capturing the characteristics of signal propagation and transmission. Specifically, we modeled the network consisting of two neurons using a simple multicompartmental formalism, with each neuron having two compartments: a ball representing a soma and a stick representing an axon. Passive and active properties of simulated neurons were adapted from existing models.^40^^,^^76^ While these parameters provide a reasonable starting point, we did not independently validate them against patch-clamp data from NGN2 neurons specifically. The modeling approach prioritized capturing the essential features observable in extracellular recordings rather than replicating all aspects of intracellular neuronal dynamics. Pre- and post-synaptic neurons were connected by excitatory synapses to study the synaptic transmission between the neurons (see methods for details). The model embodied Occam’s razor—complex enough to study synaptic transmission in this experimental system, yet simplified by omitting dendritic structure and multiple synapse locations.

**Figure 6:**
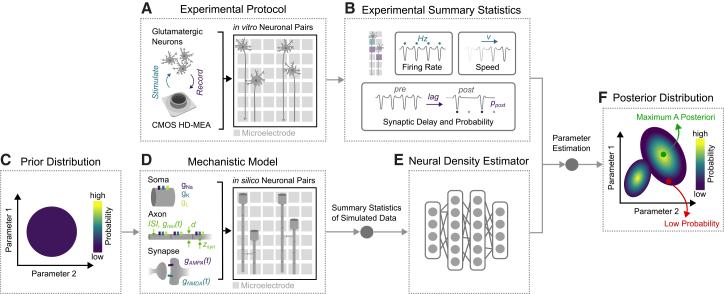
Schematic overview of the method (adapted from Gonçalves et al.^77^) (A) First, NGN2 neurons are cultured on CMOS high-density microelectrode arrays (HD-MEAs) in PDMS microstructures, enabling stimulation and recording from *in vitro* neuronal pairs. (B) Experimental summary statistics were extracted from the spontaneous activity recordings. (C) 100,000 parameter configurations were drawn from a uniform prior distribution of model parameters. (D) Using a mechanistic model of isolated neuronal pairs incorporating Hodgkin-Huxley-type neurons, these parameters drove *in silico* neuronal pair simulations. (E) A neural density estimator was trained on summary statistics from the simulated data. The trained neural density estimator was used to predict parameter estimations from experimental summary statistics. (F) Posterior distribution resulting from parameter estimation with the neural density estimator, highlighting the maximum a posteriori estimate and regions of low probability in the parameter space.

For model fitting, we used SBI.^48^^,^^49^^,^^77^^,^^78^ Instead of direct likelihood calculation that would require the ability to integrate over all potential paths through the simulator code, in SBI, forward simulations are used to train a model (typically, a neural network) to approximate parts of Bayes’ rule. Here, we used SBI to approximate the posterior distribution (Figure 6). This approach was found to be particularly useful in neuroscience, since it is possible to infer parameters in highly complex models without closed-form likelihoods.^77^ We extracted six key summary statistics (Figure 6A) from the electrophysiological recordings from neuronal pairs on MEAs (Figure 6B), including: firing rates, conduction speeds, postsynaptic spike probability, and synaptic delays. These experimental statistics defined the loss function for training a neural density estimator (NDE), ensuring the NDE identified the most likely probability distribution consistent with the provided data. To train the model, we generated a simulated dataset by sampling from uniform prior distributions over biophysically relevant parameters (Figure 6C) and feeding these into the mechanistic HH model (Figure 6D). The NDE (Figure 6E) was then trained on this simulated data to approximate the probability distribution of model parameters given specific summary statistics. Using the model trained this way, we could infer the corresponding model parameters for each recorded neuron pair, along with the corresponding posterior distribution of model parameters (Figure 6F) as a comprehensive view of the parameter space that is consistent with the observed data.

**Figure 7:**
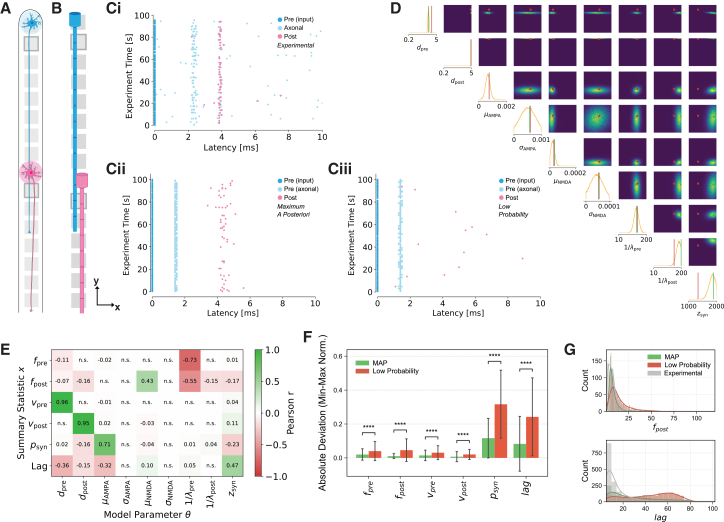
Neuronal pairs parameter inference and validation (A) Pre-synaptic (blue) and post-synaptic (pink) neurons in the experimental setup are modeled using a (B) mechanistic neuronal network model with presynaptic (blue) and post-synaptic (pink) ball and stick neurons. (C) Comparison of latency plots that were obtained experimentally and with the model. Ci) Experimental data display pre-input (dark blue), axonal (light blue), and post-synaptic (pink) spike latencies across the experimental time can be approximated with the mechanistic models using Cii) maximum a posteriori parameter configuration. The maximum a posteriori parameter configuration shows improved alignment between predicted and experimental latencies compared to Ciii) low probability parameter set simulations. (D) Inferred posterior of nine model parameters: axon diameter of the pre- and postsynaptic neuron (*d*_*pre*_, *d*_*p*_*ost*), respectively, mean and standard deviation of AMPA- and NMDA-receptor weight (*μ*_*AMPA*_, *σ*_*AMPA*_, *μ*_*NMDA*_, *σ*_*NMDA*_), respectively, intrinsic activity parameter for pre- and postynaptic neuron (1/*λ*_*pre*_, 1/*λ*_*post*_), respectively, and synapse location (*z*_*syn*_) derived from the experimental data shown in Ci). Univariate pairwise marginals are shown on the diagonals and off-diagonal, where the maximum probability parameter configuration is shown in green (dot and line), and a low probability parameter configuration example is shown in red. (E) Correlation matrix shows dependencies between six summary statistics (firing rate and conduction speed of pre- and postsynaptic neurons, respectively, postsynaptic spike probability, and the spike delay) and the nine model parameters mentioned in D. (F) Summary statistics derived from model simulations using maximum a posteriori (green) parameter configurations deviate significantly less from summary statistics derived from experimental data compared to statistics derived from model simulations using low probability (red) parameter configurations. Statistical significance indicated (∗∗∗∗*p* < 0.0001, Mann-Whitney-Wilcoxon test, for more details, check Table S6). Bar plots show mean ± standard deviation. (G) Distribution histograms comparing frequency counts between the MAP model (green), low probability model (red), and Experimental data (gray) for two example statistics. Histograms show the count of total units, with an overlaid kernel density estimate (KDE) curve.

First, we demonstrated that the experimental setup (Figure 7A) can be successfully emulated by a biophysical model (Figure 7B), whose parameters were inferred using SBI. This was first shown for one exemplary neuronal pair (Figure 7Ci) exhibiting characteristic latency patterns between pre- and postsynaptic neurons over time. Simulations using maximum a posteriori (MAP) parameter configurations replicated these experimentally observed activity patterns (Figure 7Cii), while simulations using low-probability parameter sets substantially deviated from the experimental data (Figure 7Ciii). Analysis of the full posterior distribution (Figure 7D) shows that most parameters, such as the presynaptic and postsynaptic diameters (*d*_pre_, *d*_post_), average inter-event times of spontaneous neuronal activity (1/*λ*_pre_, 1/*λ*_post_), average AMPA/NMDA synaptic weights (*μ*_AMPA_, *μ*_NMDA_), and synapse location (*z*_syn_) exhibited narrow posteriors, indicating that the experimental data strongly constrain these parameters and the recorded observables exhibit minimal redundancy or compensation from other parameters. For instance, neuronal diameters primarily determine conduction speed, which is directly captured in the experimental summary statistics. Other parameters displayed broader distributions, particularly the standard deviations of the weight of the AMPA and NMDA receptors (*σ*_AMPA_, *σ*_NMDA_), implying that multiple parameter combinations can reproduce the experimental data or that the experimental summary statistics are weakly sensitive to these parameter variations. To further understand how these parameters affect summary statistics, we analyzed correlations between summary statistics **x** and model parameters **θ** across all simulations (Figure 7E). The resulting correlation matrix revealed significant associations, with strong correlations confirming expected biophysical relationships such as the tight coupling between axon diameter and conduction speed, and AMPA receptor weight with synaptic probability.^79^^,^^80^

Using this framework, we were able to infer likely parameter values for over 1000 experimental pairs at DIV 35. The min-max normalized absolute deviation was clearly below or close to 0.1 for all summary statistics when using MAP parameters for the simulations, demonstrating the accurate reproduction of experimental features (Figure 7F), non-normalized values in Figure S11A). In contrast, simulations from low-probability parameter sets showed much larger deviations, particularly for synaptic probability and delay (lag). The smaller absolute deviation for the low probability parameter configuration in conduction speed is consistent with the narrow marginal distributions observed for the pre- and postsynaptic diameter parameters (Figure 7D), which are the main parameters determining conduction speed (Figure 7E). Additionally, we also quantified the information loss when approximating the experimental data with the models using the KL divergence (Figure S11B). The information loss using the MAP parameter configuration was lower than or equal to the low probability configuration for all parameters except the presynaptic neuron activity, which again can be explained through the dependency on parameters with narrow marginal distributions.

These quantitative findings were further supported by distribution histograms (Figure 7G) from the same set of simulated and experimental pairs, which illustrate that the MAP model aligns closely with the experimental data for both post-synaptic firing frequency and synaptic probability, whereas the low-probability model exhibits marked discrepancies. Other distribution histograms were less distinct (Figure S11C). These results confirm that SBI can robustly identify model parameters that replicate neuronal network activity as measured on MEAs.

### Neuronal model can explain synaptic potentiation upon stimulation

Previous studies in more complex preparations have demonstrated that the synchronous stimulation of pre- and postsynaptic neurons can induce activity-dependent synaptic changes, such as LTP.^9^^,^^11^^,^^14^ We sought to replicate this phenomenon in our simplified *in vitro* system and characterize the observed changes using the SBI approach to investigate the possibility of targeted modulations of synaptic strength.

**Figure 8:**
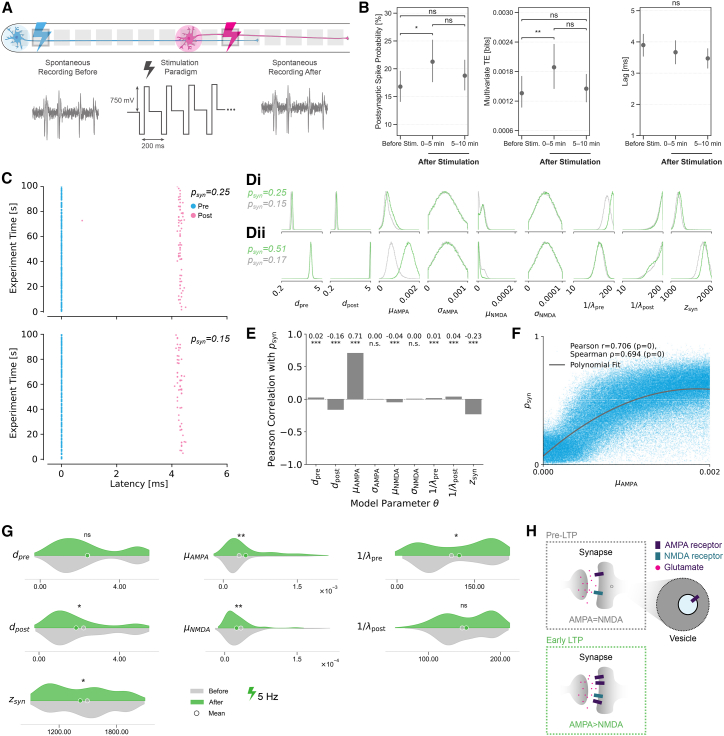
Synaptic modulation with extracellular stimulation (A) 5-min experimental stimulation paradigm consists of 5-s 5 Hz simultaneous stimulation of electrodes that belong to pre- and postsynaptic neurons with 5-s break in-between. The spontaneous activity is recorded and analyzed before and twice consecutively after stimulation. (B) Synaptic metrics quantifying changes upon stimulation. Postsynaptic spike probability and multivariate transfer entropy show significant increases immediately following stimulation, while lag remains statistically unchanged. Data are represented as mean ±95% confidence interval (∗*p* < 0.05 and ∗∗*p* < 0.01, Kruskal-Wallis omnibus tests followed by Dunn’s tests, for more details check Table S3). (C) Latency plots of the same neuronal pair before and after stimulation. The postsynaptic spike probability values are 0.15 and 0.25 before and immediately after stimulation, respectively. (D) Single sample marginal distribution changes. Di) Distribution changes for an experimental example from C). Dii) Distribution changes for a 3-fold change in *p*_*syn*_. (E) Pearson’s correlation between the postsynaptic spike probability and the model parameters computed across prior simulations. (F) Postsynaptic spike probability increases with the weight of AMPA in the HH model, reaching saturation at high values of AMPA. (G) KDE density plots of the MAP parameter configurations identified in all included neuronal pairs (*N* = 194) before and after stimulation with 5 Hz. Statistically significant differences between the means are indicated (∗*p* < 0.05, ∗∗*p* < 0.01, KS-Test, for more details check Tables S4). (H) Schematic of the AMPA and NMDA regulating synapse dynamics and stabilization (adapted from Rao et al.^81^).

We implemented a stimulation paradigm comprising three phases: an initial 5-min spontaneous recording to establish baseline activity, followed by the simultaneous stimulation of pre- and postsynaptic neurons through the identified source and target electrodes (Figure 8A), and concluding with two sequential 5-min recordings, one to capture immediate post-stimulation effects and another to quantify the potential recovery to pre-stimulation baseline conditions. Both postsynaptic spike probability and multivariate transfer entropy (see Methods) significantly increased after stimulation (Figure 8B). The two metrics quantify the temporal coupling between pre- and postsynaptic activity. Their increase despite slightly reduced spontaneous firing in both neurons (Figures S12B and S12C) demonstrates that the observed changes reflect changed synaptic transmission rather than altered excitability alone. Additionally, transfer entropy includes rigorous statistical testing against shuffled null distributions to exclude random temporal coincidence.^69^ While there is an observable decrease of both metrics in the second recording after stimulation, the values are not significantly different from either the ones before or the ones immediately after stimulation. The observed dynamics of postsynaptic spike probability and multivariate transfer entropy, with an increase in the first post-stimulus period followed by a decrease (Figure 8B; Figure S12A), could reflect either active homeostatic regulation or passive decay of transient synaptic potentiation.^11^^,^^82^^,^^83^ Whether these transient changes can consolidate into persistent modifications, and the mechanisms that would govern such consolidation, remain to be investigated with extended recordings and pharmacological interventions. The changes in the transfer entropy lag did not reach statistical significance, potentially because the temporal resolution (1 ms) could be insufficient to capture the potential shifts in the synaptic delay.

Using the HH model and the previously described SBI approach, we could decompose the changes in postsynaptic spike probability and multivariate transfer entropy into contributions from different synaptic components. Figure 8C shows an example latency plot of a neuronal pair with 10% increase after stimulation. Even in such cases when the changes in experimental postsynaptic spike probability were relatively small, the corresponding marginal parameter distributions (probability of individual parameter values after accounting for uncertainty in the remaining parameters) showed informative changes (Figure 8Di). Notably, there was a shift toward higher values for the mean value of AMPA contribution to the synaptic weight *μ*_AMPA_. For a 3-fold postsynaptic probability change, generated using the simulated data, where all found parameters for this example but the synaptic probability were kept constant, the shift in the mean value of AMPA synaptic weight is even more evident, suggesting a dose-dependent relationship between synaptic strength changes and AMPA receptor weight modulation (Figure 8Dii). In line with this, correlation analysis between changes in postsynaptic spike probability and model parameters sampled from the prior revealed that, while multiple parameters correlate with synaptic probability, it is most strongly correlated with the change in the mean value *μ*_*AMPA*_ of the AMPA receptor component in the model (Figure 8E). Furthermore, as shown in Figure 8F, the association between the two variables is nonlinear, suggesting that while initial increases in AMPA weight produce substantial enhancements in postsynaptic firing probability, the effect gradually saturates at higher AMPA values, consistent with physiological constraints on synaptic strengthening.

To further investigate the contribution of synaptic components to synaptic probability, we analyzed the distributions of the MAP model parameter configurations of the neuronal pairs before and after 5 Hz stimulation (Figure 8G), all parameters shown in Figure S12D). While we observed significant changes across multiple synaptic components following stimulation, the most notable change occurs in the mean synaptic weight of the AMPA receptor (*μ*_*AMPA*_), whose distribution becomes right-skewed after stimulation. The increased right-skewness suggests that potentiation is confined to a subset of synapses, while the majority remain weak, rather than reflecting a uniform shift across the population. Importantly, these changes were less pronounced when comparing pre-stimulation measurements with those taken 5–10 min after stimulation (Figure S12E), a period when postsynaptic spike probability is returning to baseline levels.

Some of the other parameters (*d*_*post*_, *μ*_*NMDA*_, *z*_*syn*_, 1/*λ*_*pre*_) also exhibited significant alterations following stimulation. These changes suggest more complex mechanisms related to the interplay of physiological and modeling factors. For example, the observed decrease in the NMDA receptor parameter following stimulation could be explained by several factors. First, in models of activity-dependent synaptic changes, the AMPA/NMDA ratio might be more critical than the absolute receptor values. Thus, the increase in AMPA receptor parameters may be accompanied by a relative decrease in NMDA parameters to reflect this balance rather than uniform increases. Second, as seen in Figure 7E, NMDA shows prominent correlation with postsynaptic neuron firing rate, which changes significantly immediately upon stimulation (Figure S12B). The progression of the NMDA component over days *in vitro* also shows an increasing trend (Figure S15), in line with the increasing firing rate over time.

Based on the experimental results, our 5 Hz stimulation protocol likely induced synaptic potentiation that decays over tens of minutes without consolidation. Integration with the computational model associates AMPA receptors as the main contributors to these synaptic changes. This is consistent with established mechanisms of synaptic modulation, where AMPA receptors play an important role.^80^^,^^84^ One well-studied example is early-phase LTP,^81^ where AMPA receptor modulation precedes structural changes. The pre-LTP state involves a baseline complement of AMPA and NMDA receptors, while early LTP is characterized by rapid AMPA receptor insertion and an increase in the AMPA/NMDA ratio (Figure 8H). Our modeling results align with this shift through an increased AMPA component contribution immediately upon stimulation, implying an early phase of synaptic potentiation, although identifying the underlying molecular mechanisms would require further experimental validation. The ability to directly quantify the contribution of synaptic components to synaptic potentiation in isolated neuronal pairs guides the design of further experiments.

## Discussion

*In vivo* neuronal circuits operate within highly complex environments with numerous confounding factors that often obscure fundamental synaptic mechanisms. The platform presented in this work addresses this challenge by combining the microfluidic isolation of single neuronal pairs, parallel high-throughput MEA recording from hundreds of such isolated units, human-derived NGN2-induced neurons, and integrated biophysical modeling with SBI. This combination provides a simplified experimental system where excitatory neurons form controlled connections in PDMS microstructures, allowing the direct observation of signal transmission between defined neuronal pairs while maintaining long-term viability.

We maintained hundreds of isolated parallel neuronal pairs for more than 100 days, outperforming other existing approaches^15^^,^^16^^,^^23^^,^^24^^,^^41^^,^^43^^,^^85^^,^^86^ both in terms of the recording throughput and the longevity of cultures, without the presence of support cells. While previous studies have demonstrated single-cell resolution in iPSC-derived neurons,^15^^,^^16^ autaptic cultures,^41^^,^^42^ and combined HD-MEA recordings with other techniques,^43^ our platform uniquely achieves the microfluidic isolation of many neuronal pairs in parallel. Additionally, the substrate preparation and experimental workflow require fewer steps compared to other single-cell approaches that involve more extensive preparation protocols.^23^^,^^85^^,^^87^ Several limitations of our current protocol should be considered for future development. The stochastic nature of neuronal entry into microchannels meant we lacked precise control over the number of neurons within each structure, introducing variability in experimental conditions. Additionally, NGN2-derived excitatory neurons developed extensive axonal projections that could exceed the channel length limitations imposed by CMOS chip dimensions, potentially causing undesired backgrowth. While incorporating curved channel designs could accommodate longer axons, this modification would reduce the number of replicates per chip, thereby reducing statistical power.

The reductionist approach presented here, by design, does not capture the full cellular diversity and network complexity present in brain regions *in vivo*. The current implementation uses a homogeneous population of excitatory human induced pluripotent stem cell (hiPSC)-derived NGN2 neurons to demonstrate feasibility, and presents a methodological foundation that is designed to be generalizable. The same experimental pipeline can be systematically applied to different neuronal subtypes or specific cell-type combinations of interest. The modularity in design and dimensions, as opposed to more standard microfluidic approaches,^24^^,^^88^ enables future extensions to include defined neuronal subtypes and can be scaled to incorporate more complex circuit motifs, thereby enabling a bottom-up approach to progressively build and characterize circuit complexity from well-defined cellular components. Additionally, the possibility of integration with nanochannels^22^^,^^25^ allows for subcellular analysis.

The parallel architecture and MEA compatibility enabled the collection of large electrophysiological datasets from single neurons and neuronal pairs. While synaptic transmission mechanisms have been difficult to dissect using extracellular electrophysiology alone^89^ and organoids,^90^ integrating electrophysiological recordings with biophysical modeling may provide mechanistic insights. The reduced complexity of modeling neuronal pairs, rather than large networks, allows fitting of substantial electrophysiological data without extensive computational resources. As demonstrated in the stimulation paradigm example, this integrated approach could resolve modest experimental changes (e.g., 10% increases in synaptic probability) into specific underlying synaptic parameter modifications, illustrating the potential utility of combining these techniques. This might be particularly valuable for e.g., disease models in which the affected receptor mechanisms are unknown. Unlike *in vitro*/*in silico* approaches that capture only overall culture behavior,^40^^,^^44^^,^^45^ the framework allows for the extraction of computational properties from specific neuronal pairs and the subsequent experimental investigation of the same pairs, creating a bidirectional feedback loop between modeling and experimentation.

The perturbation study presented here, while demonstrating statistically significant changes in postsynaptic spike probability, represents only an initial feasibility study into the study of synaptic transmission. For more complex electrical stimulation paradigms,^12^^,^^91^^,^^92^ one would need to account for cell-electrode positions as well as conduction and synaptic delays between identified pre- and postsynaptic pairs in more detail. If this limitation is addressed, theoretical learning rules such as Bienenstock-Cooper-Munro (BCM) learning rule^93^ or spike-time-dependent plasticity (STDP)^94^^,^^95^^,^^96^ along with its variations^92^^,^^97^ could be tested. Current understanding of plasticity mechanisms has been largely derived from computational models or inferred from population-level recordings where individual synaptic contributions cannot be disentangled. Expanding the repertoire of stimulation frequencies and stimulation delays would thus be a promising next step to bridge the gap between theoretical frameworks and empirical measurements. Importantly, emerging evidence indicates that different neuronal subtypes employ distinct plasticity mechanisms,^98^^,^^99^ yet systematic experimental comparisons across defined cell-type combinations are technically challenging, especially using human neurons. The modular design and straightforward computational integration of our platform position it to address this need, allowing researchers to systematically compare synaptic properties between specific neuronal subtypes, such as different classes of excitatory neurons or excitatory-inhibitory pairs, or between healthy and diseased neurons.

Such a more comprehensive characterization of synaptic properties would require both experimental and modeling enhancements of the current approach. Although we demonstrated functional connectivity through pharmacological validation and characterized maturation via firing rate stabilization, detailed synaptic parameters such as vesicle release probability, short-term plasticity dynamics, and presynaptic versus postsynaptic variability would require additional experimental protocols (e.g., paired-pulse stimulation, coefficient of variation analysis). Such measurements would enable corresponding enhancements to the computational model, incorporating, for example, receptor kinetics, calcium dynamics, or synaptic learning rules to generate and validate mechanistic hypotheses about synaptic maturation and potentiation.

An important consideration is that our current modeling framework does not distinguish between synaptic plasticity and intrinsic plasticity, an activity-dependent modulation of voltage-gated ion channels that alters neuronal excitability.^83^^,^^100^ Activity-dependent changes can involve both synaptic modifications (receptor trafficking, release probability) and intrinsic excitability changes occurring at both presynaptic^101^^,^^102^ and postsynaptic^100^^,^^103^^,^^104^^,^^105^^,^^106^ sites. Since the SBI approach applied here constrains synaptic parameters while holding intrinsic membrane properties fixed, any activity-dependent changes in voltage-gated conductances, spike threshold, or membrane excitability would be absorbed into the inferred “synaptic” parameters. The observed increases in postsynaptic spike probability and AMPA receptor weights following stimulation could therefore reflect genuine synaptic strengthening, enhanced postsynaptic excitability, or, most likely, a combination of both mechanisms. Distinguishing these contributions would require extending the model to include the activity-dependent modulation of intrinsic properties and/or independent experimental measurements of membrane excitability through current-clamp recordings.

Additionally, our biophysical model used parameters from healthy human iPSC-derived neurons^40^ without independent validation against the NGN2-specific patch-clamp data. While the model successfully reproduced our extracellular measurements, parameter uncertainty represents a limitation. However, our conclusions about activity-dependent synaptic changes rely on relative parameter shifts rather than absolute values, making them more robust to this limitation.

Despite the limitations mentioned above, the adaptability of our system supports numerous promising applications across neuroscience research and beyond. The platform serves as a foundation for incrementally increasing system complexity in a controlled manner, where researchers could systematically introduce additional elements—such as inhibitory neurons, specific glial populations, or defined neuromodulators—and directly observe their effects on synaptic function and information transmission. Additional applications include high-throughput drug screening with single-neuron resolution, the investigation of synaptic transmission failures and their recovery mechanisms, and systematic characterization of disease-related synaptic dysfunction in patient-derived neurons. The platform’s compatibility with optical techniques also opens possibilities for combined electrophysiological and optical studies, enabling correlation between structural and functional synaptic changes.

In conclusion, our platform enables studying the human synaptic transmission by combining synaptic pair isolation with the statistical power of high-throughput analysis. The integration of PDMS microstructures, HD-MEAs, and biophysical modeling created a versatile experimental framework that bridges the gap between reductionist approaches and biological complexity. As neuroscience increasingly demands human-relevant models for understanding brain function and dysfunction, our platform offers a scalable solution for investigating the fundamental mechanisms of neuronal communication with high detail and throughput.

### Limitations of the study

Several limitations of this study should be considered when interpreting the results. First, the stochastic nature of neuronal migration into microchannels means that precise control over the number of neurons per microchannel cannot be guaranteed, introducing variability in the experimental conditions across pairs. While the majority of channels contained one or two neurons at DIV 7, channels with more than two cells introduce ambiguity in the attribution of recorded signals to specific neurons. Second, the current platform relies exclusively on a homogeneous population of excitatory hiPSC-derived NGN2 neurons, which does not capture the cellular diversity present in native neural circuits. The absence of inhibitory interneurons, glial support cells, and neuromodulatory inputs limits the complexity of the synaptic phenomena that can be studied. Third, our biophysical model employs a simplified ball-and-stick morphology without dendritic compartments, and its passive and active parameters were adopted from existing models of NGN2 neurons without independent validation against patch-clamp recordings from the specific cell line used here. Consequently, model-inferred parameters should be interpreted as approximations consistent with the extracellular observables rather than as precise intracellular measurements. Fourth, the current modeling framework does not distinguish between synaptic and intrinsic plasticity, meaning that activity-dependent changes in voltage-gated ion channels could be partially absorbed into inferred synaptic parameters. Finally, the 5 Hz stimulation protocol used here induced only transient changes in synaptic probability that partially decayed within 10 min, and whether more persistent plasticity can be induced in this system remains to be demonstrated with extended stimulation paradigms and longer post-stimulation observation windows.

## Resource availability

### Lead contact

Requests for further information and resources should be directed to and will be fulfilled by the lead contact, Dr. Katarina Vulić (kvulic.96@gmail.com).

### Materials availability

The PDMS microstructures used in this study are commercially available from Wunderlichips (Zurich, Switzerland). The hiPSC-derived NGN2 neurons were provided by Novartis and are subject to a materials transfer agreement; requests should be directed to the lead contact.

### Data and code availability

Data and code are publicly available at the following locations.

• Data are available through the ETH Research collection: https://doi.org/10.3929/ethz-c-000797128.
• Original code has been deposited at GitHub and GitLab: information-theoretic pipeline: https://github.com/altiki/TE_code.git; recording, processing and spike plotting: https://github.com/altiki/cmos_toolbox_w_spike_sorter.git; the simulation code: https://gitlab.ethz.ch/vvasiliau/single_synapse/.
• All other items can be requested from the lead contact.

## Acknowledgments

This work was supported by 10.13039/501100003006ETH Zurich and 10.13039/501100001711Swiss National Science Foundation (Project Nr. 182779). The authors thank Dr. Julian Hengsteler for video editing and compiling. The authors further thank other LBB members for fruitful discussions.

## Author contributions

GA: conceptualization, methodology, supervision, software, validation, formal analysis, investigation, writing, and visualization. VV: conceptualization, methodology, software, validation, formal analysis, investigation, writing, and visualization. JD: conceptualization, methodology, supervision, and writing – review and editing. MLAS: validation, formal analysis, software, investigation, writing – review and editing. TS: validation, formal analysis, investigation, and writing – review and editing. AS: validation, formal analysis, investigation, and writing – review and editing. FCT: validation, formal analysis, software, investigation, and writing – review and editing. JK: validation, formal analysis, software, investigation, and writing. TR: investigation and writing – review and editing. JV: conceptualization, resources, project administration, funding acquisition, and writing – review and editing. KV: conceptualization, methodology, supervision, software, validation, formal analysis, investigation, writing, and visualization.

## Declaration of interests

The authors declare no competing interests.

## Declaration of generative AI and AI-assisted technologies in the writing process

During the preparation of this work, the authors used *Claude* in order to improve the writing style and language. After using this tool/service, the authors reviewed and edited the content as needed and take full responsibility for the content of the publication.

## STAR★Methods

### Key resources table

**Table:** 

REAGENT or RESOURCE	SOURCE	IDENTIFIER
**Antibodies**		

Rabbit anti-Synapsin	Abcam	AB254349
Mouse anti-PSD-95	Abcam	AB13552
Chicken anti-neurofilament	Abcam	AB4680
Alexa Fluor 488 goat anti-chicken	Life Technologies	A11039
Alexa Fluor 555 goat anti-rabbit	Invitrogen	A32732
Alexa Fluor 647 goat anti-mouse	Invitrogen	A32728

**Chemicals, peptides, and recombinant proteins**		

Paraformaldehyde (PFA)	Sigma-Aldrich	1004960700
B-27	ThermoFisher	17504-044
NBQX	Sigma-Aldrich	N183
AP5	Sigma-Aldrich	A8504
Matrigel	Corning	354234
Doxycycline	Clontech Labs	631311
GlutaMAX	ThermoFisher	35050-061
Penicillin-Streptomycin	ThermoFisher	15070-063
Calcein	Life Technologies	C3099
NeuO	StemCell Technologies	01801
Ethidium Homodimer	ThermoFisher	L3224 B
Hoechst 33342	ThermoFisher	H3570

**Experimental models: Cell lines**		

hiPSC-derived neurons non-labeled	Novartis	iND3 hDFa90.1.2iNgn2
hiPSC-derived neurons GFP	Novartis	iND3 hDFn83.22-iNgn2-5-AVS-CAG-GFP

**Software and algorithms**		

MaxLab	Maxwell Biosystems	v. 22
SpikeInterface	https://elifesciences.org/articles/61834	Spikeinterface 0.100.6
NEURON	https://pubmed.ncbi.nlm.nih.gov/11496923/	neuron 9.0.1
IDTxl	https://github.com/pwollstadt/IDTxl	IDTxl 1.6.0
Fluoview	Olympus	FV2000
Information metrics	https://github.com/altiki/TE_code.git	TE_code
Spike processing & plotting	https://github.com/altiki/cmos_toolbox_w_spike_sorter.git	CMOS Toolbox
Simulation code	https://gitlab.ethz.ch/vvasiliau/single_synapse/	Single Synapse

**Deposited Data**		

Data	https://doi.org/10.3929/ethz-c-000797128	Single Neuron Data

Human induced pluripotent stem cell (hiPSC) derived neurogenin-2 (NGN2) excitatory neurons were cultured inside single-cell polydimethylsiloxane (PDMS) microstructures either on top of glass substrates for visual assessment and quantification or on top of HD-CMOS MEAs for electrophysiology characterization.

### Experimental model and study participant details

All experiments were conducted with hiPSC-derived NGN2 neurons. The following types of hiPSC-derived NGN2 neurons were used: green fluorescent protein (GFP) expressing NGN2 neurons and unlabeled NGN2 neurons.

#### hiPSC origin

NGN2 neurons were generated following Giorgetti et al. protocol^107^ and transfected with doxycycline-inducible NGN2. Neuronal differentiation was induced by 3-day doxycycline exposure. Differentiated neurons were cryopreserved in aliquots of 1-8 ⋅10^6^ cells in FBS with 5% DMSO, provided by Novartis and stored in liquid nitrogen until use. As the hiPSC-derived NGN2 neurons were sourced from Novartis, cells were used as received without additional in-house validation. Furthermore, donor sex and gender information was not available, and the potential influence of these factors on the results could therefore not be assessed. More details are available in our previous work.^52^

#### hiPSC neuron seeding and maintenance

An aliquot containing iNeurons was removed from liquid nitrogen storage and rapidly thawed at 37°C. The 1 mL thawed cell solution was transferred dropwise into 4 mL of warm NBD and centrifuged for 5 minutes at 1000 rpm. After aspirating the supernatant, cells were resuspended to a concentration of 1 ⋅ 10^6^ cells per mL. The substrate was retrieved from the incubator PBS was aspirated and a sterilized PDMS frame (1 × 1 × 1 cm^3^) was positioned on top of the microstructure. Approximately 50 *μ*L of warm medium was pipetted into the frame. The volume containing 50,000 cells was then pipetted into the frame and the dish was placed in the incubator. After approximately 20 minutes, an additional 500 *μ*L of warm medium was added around the frame to prevent excessive evaporation. The frame was removed the following day, and the medium was exchanged and topped up to approximately 1 mL.

The cells were cultured in NeuroBasal medium (NB) (21203-049, ThermoFisher).^108^ Fresh NB complete medium was prepared, consisting of a 2% solution of B-27 supplement (17504-044), a 1% solution of Penicillin-Streptomycin (P-S) (15070-063), and a 1% solution of GlutaMAX (35050-061), all sourced from ThermoFisher. In the first 10 days, doxycycline (Clontech Labs 3P 631311, Fisher Scientific) was added in a 1:500 ratio to further enhance neuronal differentiation and maturation.

### Method details

#### PDMS microstructures

PDMS microstructures for isolating and guiding single cells were designed using CAD software (AutoCAD 2021) and fabricated by Wunderlichips (Zurich, Switzerland). More details on the fabrication process are available in previous publications.^22^^,^^25^? The PDMS microstructures were fabricated with openings of 10 *μ* m (see schematic in Figure 1A). The microchannels were designed with two distinct heights: 2 *μ*m for structures containing submicrometer features and 4 *μ* m for those without, while maintaining a consistent width of 10 *μ* m across all channels (see schematic in Figures 1A and 1B). The total thickness of the PDMS microstructures was 75 *μ* m. To accommodate axonal growth, the microchannels were designed with variable lengths, all exceeding 2.5 mm, and terminated in a loop structure to entrap the extending axons. Each well was optimized to house a single neuron, with the total number of wells varied based on the specific application requirements. A photo of the PDMS membrane with visible channel and well contours is shown in Figure 1Aiii).

#### Substrate preparation and surface functionalization

The PDMS microstructures were placed either on top of glass substrates (35 *μ*m Ibidi, 81218-200-IBI, Vitaris, Switzerland) for visual assessment and quantification or on top of high-density complementary metal oxide semiconductor microelectrode arrays (HD-CMOS MEAs) for electrophysiology characterization. Before mounting, both the microstructure and the substrate were cleaned and activated in a Tergeo Plasma Cleaner (Pie Scientific, USA) using an O_2_ + H_2_O mixture for 1 minute. For CMOS chips, care was taken to leave a portion of the reference electrode uncovered during mounting. After a 2-minute waiting period to ensure initial attachment, the surfaces were functionalized with matrigel (Corning matrigel Basement Membrane Matrix, 354234, 10.5 mg/ml) diluted to 3 mg/ml in neurobasal medium. The matrigel solution was prepared 1-12 hours before use and maintained at 4°C to prevent premature crosslinking. Approximately 2.5 *μ*L of the cold matrigel solution was carefully dispensed over the microstructure openings and guided through the microchannels via gentle pipetting. Excess matrigel was removed by aspiration, and the substrates were incubated at 37°C for 5 minutes. To minimize overgrowth, an optional second plasma treatment (2 minutes, O_2_ + H_2_O) was performed to selectively remove matrigel from the top surface of the microstructure. The devices were then filled with phosphate-buffered saline (PBS) and desiccated for approximately 5 minutes to remove any air bubbles. Finally, the PBS was replaced with 500 *μ*L of complete neurobasal medium.

#### Staining and imaging

iNeurons were imaged on day *in vitro* (DIV) 7 as live cultures to assess channel occupancy and culture viability. Following neuronal maturation and synapse formation, selected samples were subsequently fixed, immunolabeled, and visualized for further analysis.

##### Live and dead cell staining

The growth of cells in single-cell PDMS microstructures was monitored using NeuroFluor^TM^ NeuO (01801, StemCell Technologies, Switzerland). NeuO is a membrane-permeable fluorescent probe that selectively labels primary and iPSC-derived neurons. On the day of imaging, cells were stained following the standard protocol provided by the manufacturer. Some cultures were stained with Calcein and Ethidium Homodimer at a 1:1000 ratio.

##### Immunofluorescence staining

The samples were fixed in the fourth or fifth week in culture using 4% paraformaldehyde (PFA) (1004960700, Sigma-Aldrich) for 15 min at room temperature. Following fixation, samples were washed three times with PBS, allowing 10 min intervals between washes. Cell membranes were then permeabilized using PBS containing 0.1% Triton X-100 (X100, Sigma-Aldrich) for 5-8 minutes, after which the permeabilization solution was removed by three consecutive PBS washes with appropriate waiting periods. Subsequently, samples were incubated with primary antibody solution containing goat serum (31873, Invitrogen) and the following primary antibodies at 1:1000 dilution: rabbit anti-Synapsin (AB254349, Abcam), mouse anti-PSD-95 (AB13552, Abcam), and chicken anti-neurofilament (AB4680, Abcam). To enhance antibody diffusion into the PDMS microchannels, samples were incubated for 48 h on a shaker at room temperature. Following primary antibody incubation, samples were washed three times with PBS (10 min per wash) and subsequently incubated with secondary antibody solution consisting of Alexa Fluor 488 goat anti-chicken (A11039, Life Technologies), Alexa Fluor 555 goat anti-rabbit (A32732, Invitrogen), and Alexa Fluor 647 goat anti-mouse (A32728, Invitrogen) antibodies diluted 1:500 in goat serum. Secondary antibody incubation was also performed for 48 h on a shaker at room temperature. After incubation, samples were washed three times with PBS (10 min intervals) and counterstained with Hoechst (1:500 in PBS) for approximately 1 h. Finally, samples underwent three additional PBS washes and were stored at 4°C until imaging, which was performed either on the same or the following day.

##### Image acquisition and analysis

Cells cultured in PDMS microstructures on glass substrates were visualized using a confocal laser scanning microscope (CLSM) (Fluoview 3000, Olympus). For live cell imaging, either a 10× (Olympus, UPLFLN10X2PH, NA = 0.3) or 20× (Olympus, UPLFLN20XPH, NA = 0.5) objective was employed. To enable visualization of synaptic puncta in immunostained samples, imaging was performed using a 60× oil-immersion objective (Olympus, UPLSAP060XS2, NA = 1.3). Microscope images were analyzed using Fiji software.^109^ To improve axonal visibility relative to soma, a pixel logarithm transformation was applied to all representative fluorescent images presented in this paper, with the exception of immunofluorescent staining images. Background fluorescence was minimized through manual adjustment of brightness and contrast parameters.

#### Electrophysiology

##### Microelectrode arrays for recording and modulating neuronal activity

The experiments utilized high-density complementary metal-oxide-semiconductor (HD-CMOS) microelectrode arrays (MEAs) from Maxwell Biosystems (Switzerland). These chips (MaxOne+ Chip (uncoated Pt-electrodes)) feature a flat surface topology and incorporate 26,400 electrodes arranged across a 3.85 x 2.10 mm^2^ area, with electrodes spaced at 17.5 *μ*m intervals. Through a switch matrix, any 1,020 electrodes can be selected for simultaneous recording, with data acquisition performed at 20 kHz. The system also enables extracellular stimulation through the same electrodes using 32 independent stimulation buffers.

##### Visualization of PDMS microstructure with a CMOS MEA

A custom Python script was used to generate a voltage map displaying the positions of electrodes beneath the PDMS microstructure. An example of this voltage map for the PDMS microstructure is illustrated in Figure S13. This mapping enables precise identification and selection of electrodes that correspond to individual networks within the PDMS microstructure. A detailed description of this methodology can be found in our previous publication.^26^

##### Recording area selection

Upon first recording, the total chip area was split into separate electrode subselections that contained no more than 1,020 electrodes (upper limit for the number of electrodes recorded simultaneously). This number ranged from 6 to 11 areas, depending on the voltage map (PDMS placement quality).

##### Data collection

The combination of subselections of the recording area with PDMS microstructures facilitated repeated recordings of identical areas, and consequently the same neurons, over time. For recording sessions, MEA chips were inserted into the recording units, which were placed in an incubator maintained at 35 °C, 5 % CO_2_, and 90 % humidity. Following placement of the culture in the incubator, a 5-minute settling period was implemented to allow CO_2_ levels and humidity to stabilize after door opening and to permit culture equilibration. Spontaneous neuronal activity was subsequently recorded for 2-5 minutes per subselected area at a sampling frequency of 20 kHz. Recordings were conducted approximately weekly during the first five weeks in culture and less frequently at later time points, with the most extended recording occurring at DIV 234 (Figure S14).

##### Chemical suppression of synapses

Cultures at DIV 100 were treated with the synaptic blockers (6-nitro-7-sulfamoylbenzo(f)quinoxaline-2,3-dione) NBQX and AP5, which act as selective antagonists for AMPA and NMDA receptors, respectively. Each antagonist was diluted in NBD to 10 *μ*M. Spontaneous activity was first recorded during two consecutive 2-minute periods for each selected chip area to establish baseline measurements. Following baseline recording, both blockers were added to the culture medium, and spontaneous activity was immediately measured for three consecutive 2-minute periods in each area to capture the acute effects of synaptic inhibition. The culture medium was then aspirated and replaced with fresh NBD medium three times to ensure complete washout of the synaptic blockers. To assess potential long-term effects, spontaneous activity was recorded again for 2 minutes in each area one week after treatment.

##### Stimulation paradigm

Stimulation experiments shown in Figure 8 were performed on DIV 32, 35, 41, 46. The stimulation paradigm consisted of 5-min spontaneous recording which was followed by a 5-min stimulation. Stimulation consisted of 5-second 1.5 V peak-to-peak (Vpp) stimulation pulses (biphasic, first pulse negative, total pulse duration 400 *μ*s) delivered at a 5 Hz frequency, interchanged with 5 second break time. Two 5-min spontaneous recordings were performed sequentially after stimulation. The stimulation parameters were selected based on our previous experiments using similar systems, where 5 Hz and 1.5 Vpp stimulation evoked responses in stimulated neurons and secondary responses in their synaptic partners.^110^ We also performed synaptic modulation experiments at additional frequencies (Figure S16), but achieved less robust results.

#### Data analysis: Neuronal pair identification and synaptic pair calculation

Data was processed and analyzed using a custom-made data analysis pipeline. First, spikes were detected and sorted using the Spikeinterface framework^111^ including SpyKING CIRCUS v2 spike sorter.^112^ The spike detection parameters included detection at a negative peak exceeding 5 times the signal standard deviation and 3 ms minimum spike distance. Identified spike units were excluded if the signal-to-noise ratio was below 5 or the firing rate of the spike unit was below 0.1 Hz. The following metrics were calculated per unit using custom-made scripts: firing rate, conduction speed, and waveform metrics: amplitude, peak-trough ratio and duration, half width, repolarization slope and recovery slope.

To identify putative synaptic pairs *i.e.*, synaptic coupling between units, multivariate transfer entropy (mTE) was used. mTE is an information-theoretic measure that quantifies the directed transfer of information between multivariate time series. It extends the concept of transfer entropy by accounting for the influence of multiple variables on the information flow. Consider two stochastic processes X and Y. Each stochastic process is a collection of random variables {*X*_*t*_}, where for each variable its realized value is denoted by *x*_*t*_. We will also use length-*ℓ* and length-*k* collections of random variables (embedding vectors) $Y_{t-\delta }^{\left(\ell \right)}=\left\{Y_{t-\delta -\ell },Y_{t-\delta -\ell +1},\ldots ,Y_{t-\delta }\right\}$ and $X_{t-1}^{\left(k\right)}=\left\{X_{t-k},X_{t-k+1},\ldots ,X_{t-1}\right\}$, where *δ* is the time delay. Transfer entropy from Y to X is defined as the reduction in uncertainty about the future state of X when considering the past states of Y, over and above what is already available in the past states of X,^66^^,^^67^^,^^113^ expressed in the form of conditional mutual information:

(Equation 1) $$\begin{array}{c} T_{Y\to X}^{\left(k,\ell ,\delta \right)}\left(t\right)\equiv I\left(X_{t};Y_{t-\delta }^{\left(\ell \right)}|X_{t-1}^{\left(k\right)}\right)=\\ H\left(X_{t}|X_{t-1}^{\left(k\right)}\right)-H\left(X_{t}|X_{t-1}^{\left(k\right)},Y_{t-\delta }^{\left(\ell \right)}\right), \end{array}$$

where *I*(*X*; *Y*) is mutual information between the two random variables X and Y that quantifies the reduction in uncertainty (entropy) about one variable given knowledge of the other.

mTE generalizes Equation 1 to account for the influence of other potential sources. This is crucial for distinguishing direct from indirect information transfer, especially in networks where multiple units may be interconnected or driven by common inputs. mTE from Y to X is conditioned on a collection of other stochastic processes **Z**:

(Equation 2) $$T_{Y\to X|Z}^{\left(k,\ell ,\delta ,m\right)}\left(t\right)\equiv I\left(X_{t};Y_{t-\delta }^{\left(\ell \right)}|X_{t-1}^{\left(k\right)},Z_{t-1}^{\left(m\right)}\right),$$

where $Z_{t-1}^{\left(m\right)}$ denotes the past of the conditioning set (collection of stochastic processes) and *m* is the corresponding embedding length.

Inference of the functional connectivity using mTE was done using IDTxl.^114^ The IDTxl software utilizes an optimization approach that combines greedy search algorithms with comprehensive null hypothesis testing to determine the optimal set of parent nodes **Z** and historical embeddings for both the past of X and Y.

Despite the optimization, computational demands for mTE calculation remain large: complete network analysis has a computational complexity of *O*(*N*^2^ × *d* × *τ*_max_ × *S*), where *N* represents neuron count, *d* indicates average incoming connections per neuron, *τ*_max_ denotes maximum temporal search depth, and *S* represents the number of surrogate tests.^115^^,^^116^ Due to these computational constraints, a preselection step was implemented to reduce the number of potential incoming connections to each neuron as previously described in Varley et al.^115^ The incoming source nodes were prefiltered for each target neuron by only including neurons that demonstrated statistically significant bivariate transfer entropy (Equation 1) to the target neuron across temporal search depth *τ*_max_ of 10 ms for the source and 5 ms for the target. Statistical significance was evaluated using the empirical null hypothesis test, implemented in JIDT software package.^117^ The null distribution was inferred using 200 permutations. False discovery rates were corrected for using the Benjamini-Hochberg correction for multiple testing (*α* = 0.05).^118^ Significant source-target pairs as identified with bivariate transfer entropy were used to compute multivariate transfer entropy (Equation 2) using IDTxl. Following other work on neuronal data,^115^^,^^119^^,^^120^ only one bin of source history was considered, defined as the lag that maximized the significant bivariate transfer entropy. On the other hand, the target history was searched using a search depth *τ*_max_ of 10 ms.

Once synaptic pairs were identified, defined as source and target neurons with a significant multivariate transfer entropy (p ≤ 0.05), the probability of postsynaptic spike was calculated as follows:

(Equation 3) $$P_{\mathrm{syn}}=\frac{N_{\mathrm{output}}\left(t\in \left[lag-\Delta t,lag+\Delta t\right]\right)}{N_{\mathrm{input}}},$$

where Δt was set to 1 ms. Pairs with a postsynaptic spike probability greater than 0.05 were kept for further analysis.

#### Data analysis: Prediction of firing probability with machine learning models

To predict whether a postsynaptic spike occurs within a defined time window, we trained a transformer model *f*(⋅; *θ*) on cutouts of extracellular voltage traces. The aim of this analysis is to evaluate whether the neuron pairs identified through the transfer entropy approach carry sufficient information in their prior and source (presynaptic neuron) signals to enable reliable prediction of postsynaptic spiking probability. This serves two purposes: first, to validate that the variables used in the transfer entropy analysis indeed reflect a directional information exchange—without which a predictive mapping would be impossible; and second, to gain insight into which signal components are more informative for postsynaptic spiking, thereby shedding light on the temporal structure of the underlying interaction. Pre- and postsynaptic neuron pairs were selected such that each neuron’s extremum electrode appeared only once in the dataset. The timing of events for sample extraction was based on spike times of the presynaptic neuron, adjusted by the most significant lag obtained from the transfer entropy analysis:

(Equation 4) $$e_{i}^{k}=s_{\text{pre},i}^{k}+{\text{lag}}_{k}$$

where $s_{\text{pre},i}^{k}$ is the *i*-th spike time of the presynaptic neuron in pair *k*, and lag_*k*_ is the transfer entropy lag for that pair.

From each event time $e_{i}^{k}$, fixed-length signal snippets are extracted:

(Equation 5) $$\begin{array}{cccc} Y_{i}\left[t\right] & =R_{\mathrm{pre}}^{k}\left[e_{i}^{k}+t-\Delta_{\text{source}}\right], & & t\in \left[0,w_{\text{source}}\right]\\ X_{i}\left[t\right] & =R_{\mathrm{post}}^{k}\left[e_{i}^{k}+t-\Delta_{\text{prior}}\right], & & t\in \left[0,w_{\text{prior}}\right] \end{array}$$

Here, *R*^*k*^ denotes the extracellular recording from the corresponding extremum electrode, and Δ and *w* denote the shift and window size for each signal. Context signals for *Y*_*i*_ and *X*_*i*_ are generated by selecting spike times within the same windows and convolving them with a Gaussian kernel (standard deviation 0.5 ms). The binary target *S*_*i*_ is set to one if a spike from the postsynaptic neuron occurs within a bin of width *b* centered at $e_{i}^{k}$, and zero otherwise. To form the input to the model, *n* samples are concatenated, and the model is trained to predict the mean of the corresponding *n* target values. Data was split into training (70%), validation (15%), and testing (15%) sets. Prior to the split, all input-target pairs were randomly permuted. Precise parameter values are given in Table S7.

The methodology and results are summarized in Figure S17. The model consistently outperforms a baseline predictor that uses the mean firing probability from the training data. While the model’s performance is not perfect-as extracellular recordings, though informative, do not capture synaptic currents or the inherent noise in neuronal firing-the results clearly demonstrate that both source and prior signals contain predictive information about the postsynaptic spike. Among these, the source signal contributes more significantly, suggesting a stronger influence on the postsynaptic neuron’s firing probability.

#### Computational model

*In silico*, a network is modeled as two synaptically connected multicompartmental neurons, as well as extracellular electrodes that capture the electrical field generated by the traveling action potentials. The model is implemented using LFPy library,^121^ the model of a neuron and a synapse are defined using NEURON simulator.^122^ The model’s parameters and variables are summarized in Table 1.

**Table 1:** Fixed and variable parameters of the Hodgkin-Huxley biophysical model used for simulation-based inference

Parameter	Value/Range	Type	Explanation
**Biophysical properties**			

*r*_a_	100 Ω cm	fixed	specific axial resistance
*c*_m_	1 μF/cm^2^	fixed	specific membrane capacitance
*g*_Na_	0.05 S/cm^2^	fixed	maximal sodium conductance
*g*_K_	0.005 S/cm^2^	fixed	maximal potassium conductance
*g*_L_	0.0003 S/cm^2^	fixed	leak conductance
*T*	310 K (37°C)	fixed	temperature

**Reversal potentials**			

*E*_L_	−39.2 mV	fixed	leak reversal potential
*E*_Na_	70 mV	fixed	sodium reversal potential
*E*_K_	−80 mV	fixed	potassium reversal potential
*V*_T_	−30.4 mV	fixed	threshold shift for sodium activation

**Morphology**			

*d*_soma_	10 μm	fixed	soma diameter
*ℓ*_soma_	10 μm	fixed	soma length
*ℓ*_axon_	2000 μm	fixed	axon length
*d*_axon_^pre^, *d*_axon_^post^	[0.2, 5] μm	variable	axon diameters of pre- and post-synaptic neurons

**Electrode geometry**			

(*x*_e1_, *y*_e1_, *z*_e1_)	(0, 0, 60) μm	fixed	electrode 1 position
(*x*_e2_, *y*_e2_, *z*_e2_)	(0, 0, 1060) μm	fixed	electrode 2 position

**Synaptic properties**			

*z*_syn_	[1000, 2000] μm	variable	synapse coordinate
μ_AMPA_	[0.0, 0.002]	variable	mean value of AMPA synaptic weight
σ_AMPA_	[0.0, 0.001]	variable	standard deviation of AMPA synaptic weight
μ_NMDA_	[0.0, 0.0002]	variable	mean value of NMDA synaptic weight
σ_NMDA_	[0.0, 0.0001]	variable	standard deviation of NMDA synaptic weight
τ_2_^AMPA^, τ_1_^AMPA^, ε_AMPA_	(0.1 ms, 0.3 ms, 0 mV)	fixed	timescales and reversal potential of AMPA receptors
τ_2_^NMDA^, τ_1_^NMDA^, ε_NMDA_	(10 ms, 30 ms, 0 mV)	fixed	timescales and reversal potential of NMDA receptors

**Spontaneous input current I**_**spont**_			

λ_pre_, λ_post_	[5, 100] Hz	variable	Poisson process rate
τ_1_, τ_2_, ε	0.2 ms, 0.4 ms, 0 mV	fixed	timescales and reversal potential of I_spont_
μ_spont_	0.02	fixed	mean value of spontaneous current
σ_spont_	0.02	fixed	standard deviation of spontaneous current

Parameters are grouped by category. Variable parameters were sampled during simulation; fixed parameters were held constant across all simulations.

##### Morphology

As stated before, a single neuron is modeled using multicompartmental formalism. The morphology is of a simple ball-and-stick neuron, with two compartments: a soma and an axon. Because the experimental readout relies exclusively on extracellular recordings of action potential propagation and does not directly measure subthreshold postsynaptic potentials or dendritic integration, dendritic compartments were excluded from the model to ensure that all modeled parameters are experimentally constrained. The soma is comprised of a single segment, modeled as a cylinder with the height *h* and the diameter *d* of 10*μm*. The axon is modeled as a series of cylindrical segments, each 19.9 *μm* long, with a total of 100 segments. Within a given simulation, all axonal segments share a fixed diameter *d*. Across simulations, the diameter is varied in the range 0.2 *μm* ≤ *d* ≤ 5 *μm* in increments of 0.2 *μm*.

##### Geometry

The presynaptic neuron’s soma is positioned vertically along the *z*-axis, extending from *z* = 0 *μm* to *z* = 10 *μm*. The axon originates at the distal end of the soma and extends further along the *z*-axis up to *z* = 2000 *μm*. All 100 axonal segments are aligned along this axis, resulting in a straight axon oriented in the *z*-direction. The postsynaptic neuron is positioned identically but shifted along the *z*-axis, with its soma extending from *z* = 1000 *μm* to *z* = 1010 *μm*. Its axon originates at *z* = 1010 *μm* and extends to *z* = 3000 *μm*.

##### Membrane potential

All compartments were assigned passive and active membrane properties. The specific axial resistivity was set to *r*_*a*_ = 100 Ω ⋅cm and the specific membrane capacitance to *c*_*m*_ = 1 *μ*F/cm^2^. For a cylindrical segment *n* of length *L* and diameter *d*, with membrane potential *V*_*n*_ and axial neighbors *V*_*n*−1_ and *V*_*n*+1_, the membrane potential evolves according to the cable equation^123^:

(Equation 6) $$c_{m}\frac{dV_{n}}{dt}=\frac{d}{4r_{a}l^{2}}\left(V_{n-1}-2V_{n}+V_{n+1}\right)+\frac{I_{\text{spont,n}}\left(t\right)+I_{\text{syn},n}\left(t\right)}{\pi dh}-\underset{\text{ion}}{\sum }i_{\text{ion}}\left(V_{n},t\right),$$

where *i*_ion_ denotes the ionic current density, *I*_spont_ is a current, applied to neurons to elicit spontaneous activity and *I*_syn_ is a synaptic current.

##### Ionic current densities

Ionic current densities are modeled using Hodgkin-Huxley formalism with expressions for the rate constants well suited for cortical pyramidal neurons as described previously.^40^^,^^76^ The model includes fast sodium (*i*_*Na*_), delayed-rectifier potassium (*i*_*K*_), and leak (*i*_*L*_) currents, defined as

$$\begin{array}{ll} i_{Na} & =g_{Na}m^{3}h\left(V-E_{Na}\right)\\ i_{K} & =g_{K}n^{4}\left(V-E_{K}\right)\\ i_{L} & =g_{L}\left(V-E_{L}\right), \end{array}$$

with values for reversal potentials *E* and maximal conductances *g* taken from Traub et al.^76^

Gating variables *x* ∈ {*m*, *h*, *n*} evolve according to:

$$x_{\infty }\left(V\right)=\frac{\alpha_{x}\left(V\right)}{\alpha_{x}\left(V\right)+\beta_{x}\left(V\right)},\;\tau_{x}\left(V\right)=\frac{1}{\phi \cdot \left(\alpha_{x}\left(V\right)+\beta_{x}\left(V\right)\right)},$$

where $\phi =3^{\frac{T-37}{10}}$ is a temperature-dependent scaling parameter.

Rate functions for each gating variable follow equations from Doorn et al.^40^:

$$\begin{array}{llll} \alpha_{m}\left(V\right) & =\frac{0.32\cdot \left(13-V+V_{T}\right)}{\exp\left(\frac{13-V+V_{T}}{4}\right)-1}, & \beta_{m}\left(V\right) & =\frac{0.28\cdot \left(V-V_{T}-40\right)}{\exp\left(\frac{V-V_{T}-40}{5}\right)-1}\\ \alpha_{h}\left(V\right) & =0.128\cdot \exp\left(\frac{17-V+V_{T}}{18}\right), & \beta_{h}\left(V\right) & =\frac{4}{1+\exp\left(\frac{40-V+V_{T}}{5}\right)}\\ \alpha_{n}\left(V\right) & =\frac{0.032\cdot \left(15-V+V_{T}\right)}{\exp\left(\frac{15-V+V_{T}}{5}\right)-1}, & \beta_{n}\left(V\right) & =0.5\cdot \exp\left(\frac{10-V+V_{T}}{40}\right). \end{array}$$

Here, *V*_*T*_ serves as a global voltage offset, uniformly shifting the voltage dependence of all rate functions. In Doorn et al.,^40^ Nernst potentials and a global voltage offset were adjusted to match the average resting membrane potential, spike threshold, and action potential amplitude of NGN2 neurons observed experimentally, and we adopted the same values in our simulations.

##### Synaptic currents

External and synaptic currents are assumed to be inputs from conductance-based chemical synapses. Their currents can be modeled analogously to how ion channels are modeled. For synaptic input, we have

$$I_{\text{syn}}=G_{\text{syn}}\left(t\right)\left(V-E_{\text{syn}}\right),$$

and an analogous equation for external input. The synaptic conductance *G*_syn_ is measured in millisiemens (*mS*). To model the activation of postsynaptic neurotransmitter-gated ion channels, we use a *β*-function profile that captures the rise and decay dynamics of synaptic input following activation. Assuming the synapse is activated at *t* = 0, the conductance evolves as:

(Equation 7) $$G_{\text{syn}}\left(t\right)=w_{\text{syn}}\;\Theta \left(t\right)\left(\frac{e^{-t/\tau_{1}}-e^{-t/\tau_{2}}}{e^{-\tau_{\text{peak}}/\tau_{1}}-e^{-\tau_{\text{peak}}/\tau_{2}}}\right),$$

(Equation 8) $$\text{where}\;\tau_{\text{peak}}=\frac{\tau_{1}\tau_{2}}{\tau_{2}-\tau_{1}}\log\left(\frac{\tau_{2}}{\tau_{1}}\right),$$

and Θ(*t*) is the Heaviside step function, defined as Θ(*t*) = 1 for *t* ≥ 0 and Θ(*t*) = 0 otherwise. Here *τ*_2_ is the rise and *τ*_1_ is the decay time constants of the synapse.

The inherent stochasticity of synaptic transmission observed in biological neurons, arising from probabilistic vesicle release, fluctuating neurotransmitter concentrations, and variability in postsynaptic receptor activation, was accounted for by sampling the synaptic weight *w*_syn_ (i.e., the peak synaptic conductance) at each presynaptic activation from a truncated normal distribution T with specified mean *μ* and standard deviation *σ*.

To elicit spontaneous activity in each neuron, the initial axon segment receives an excitatory synaptic input *I*_spont_ with reversal potential *E*_spont_ = 0 mV, and synaptic time constants *τ*_1_ = 0.2 ms and *τ*_2_ = 0.4 ms. The synaptic weight for a neuron with axon diameter *d* is sampled from a truncated normal distribution T(μ⋅d,σ⋅d), with *μ* = 0.02 *μ*S/*μ*m and *σ* = 0.02 *μ*S/*μ*m. Spike times are drawn from a Poisson process with rate *λ*.

The synaptic weight was scaled with compartment diameter to ensure that the synaptic efficacy is independent of compartment size. Without this adjustment, a fixed conductance input would produce smaller voltage changes in larger compartments due to their increased membrane capacitance and leak conductance. Since the compartment length is fixed across simulations, scaling with diameter alone is sufficient to normalize synaptic impact.

The synaptic connection between the two neurons is also modeled using the same formalism, i.e. using Equation 7, and drawing synaptic weights from a normal distribution with specified mean and standard deviation. *I*_syn_ was further split into AMPA component with fast time constants, and NMDA with slow time constants listed in Table 1. The rise and fall timescales for the AMPA component of the synapse were taken to be almost instantaneous, whereas for the NMDA component, these values were taken from literature.^124^ The reversal potential value for both components was also taken from literature.^124^ The synapse is located at (0, 0, *z*_syn_) and connects two compartments of the postsynaptic neuron and the postsynaptic neuron, closest to that coordinate.

##### Extracellular electrodes

Two recording electrodes placed in the extracellular space at (0, 0, 60)*μm* and (0, 0, 1060)*μm* are modeled to study the extracellular electric fields generated by neuronal activity. The extracellular medium is assumed to be homogeneous and isotropic, with a conductivity of 0.3 S/m. The potential at each recording point is computed using the line-source approximation.

#### Model fitting

##### Simulation-based inference

Simulation-based inference (SBI) was employed to estimate parameters of a mechanistic neuronal model from observed features of simulated data. Unlike traditional methods that rely on closed-form likelihoods, SBI uses forward simulations to approximate components of Bayes’ rule—such as the posterior, likelihood, or likelihood ratio—using a flexible neural network trained on simulation data.^48^^,^^77^

To approximate the posterior distribution *p*(***θ***∣**x**), which represents the probability that a given parameter set ***θ*** produced observed data **x**, a neural network *q*_*F*(**x**,*ϕ*)_(***θ***) was trained on simulated (***θ***, **x**) pairs, where *F* is a parametric family of densities and *ϕ* are its parameters. Because the training data are generated independently of any specific observation **x**_*o*_, the network learns to map arbitrary inputs to approximate posteriors, enabling *amortized inference*—a setting where inference can be rapidly performed for any new observation within the support of the prior.

##### Summary features

The inference network was trained on summary statistics extracted from simulated voltage traces and corresponding spike times, focusing on biologically meaningful features relevant to synaptic transmission. This approach reduces the dimensionality of the data and targets the aspects most constrained by experimental measurements. As summary statistics, the firing rates and conduction velocities of both neurons were used, as well as the synaptic transmission probability and the coupling lag.

Firing rates of the presynaptic (*f*_pre_) and postsynaptic (*f*_post_) neurons were defined as the number of somatic spikes per neuron divided by the total simulation duration. Conduction velocities (*v*_pre_, *v*_post_) were computed by detecting spike times at two spatially separated sites along each axon, calculating the average time delay between matched spikes, and dividing the physical distance by this latency. For the presynaptic neuron, spikes were recorded between (0, 0, 200) *μ*m and (0, 0, *z*_syn_ − 100) *μ*m; for the postsynaptic neuron, between (0, 0, *z*_syn_ + 100) *μ*m and (0, 0, 3000) *μ*m, ensuring that only forward-propagating action potentials were considered. Conduction speed was computed by dividing the temporal delay between spike detections by the corresponding axial distance between recording locations, thereby explicitly accounting for the different path lengths traveled in the pre- and postsynaptic segments.

To compute the synaptic transmission probability, the optimal coupling lag that maximized transfer entropy (as described above) was identified first, then the number of postsynaptic spikes occurring within ± 0.5 ms of that lag were counted and normalized by the total number of presynaptic spikes.

##### Inference details

SBI was implemented using the sbi library,^125^ employing neural posterior estimation with a mixture density network^126^ as the density estimator. The network was trained to minimize the negative log-likelihood of the posterior predictions over *N* simulated samples, $-\frac{1}{N}\sum_{j=1}^{N}\ln q_{F\left(x_{j},\phi \right)}\left(\theta_{j}\right)$, using the Adam optimizer with default hyperparameters.^77^ The prior distribution was defined as a uniform distribution over parameter ranges summarized in Table 1. The maximum a posteriori (MAP) estimate was obtained by drawing 10,000 samples from the learned posterior, selecting the 200 with the highest log-posterior values, and performing gradient-based optimization from each to identify the parameter value with the highest posterior density.

The network was trained on a total of 122,000 simulations, each lasting 10 s with a temporal resolution of 0.1 ms. Only simulations for which transfer entropy indicated a significant interaction (*α* = 0.05) were used for inference.

### Quantification and statistical analysis

All statistical analyses were performed in Python. The sample size N refers to the number of synaptic pairs, neurons, or chips as specified in the figure legends and supplemental tables. Statistical comparisons between two groups were performed using the Mann-Whitney-Wilcoxon test or Student’s t-test, and distribution differences were assessed using the Kolmogorov-Smirnov (KS) test. For comparisons across three or more groups, Kruskal-Wallis omnibus tests were performed, followed by Dunn’s post-hoc tests with Benjamini-Hochberg correction for multiple comparisons. Statistical significance is denoted as: ns (p > 0.05), ∗ (0.01 < p ≤ 0.05), ∗∗ (0.001 < p ≤ 0.01), ∗∗∗ (0.0001 < p ≤ 0.001), and ∗∗∗∗ (p ≤ 0.0001). Pearson and Spearman correlation coefficients were calculated to assess relationships between model parameters and summary statistics. Comparisons of forward directionality across PDMS microstructure types were performed using a one-way ANOVA followed by pairwise t-tests with Bonferroni correction for multiple comparisons. Directed functional connectivity between neuronal pairs was assessed using multivariate transfer entropy, with statistical significance evaluated against surrogate distributions using 200 permutations and false discovery rate correction applied using the Benjamini-Hochberg method (*α* = 0.05). Line plots show mean ± 95% confidence interval. Box plots show median, interquartile range, and 1.5× IQR whiskers. Violin plots show the distribution of data with median and interquartile range. Histograms show either the distribution of values as percentage of total units or the total count as indicated in the figure legend with an overlaid kernel density estimate curve. Bar plots show the mean ± standard deviation. Detailed statistical results for all comparisons are provided in Tables S1, S2, S3, S4 and S5.

## References

[1] Citri A., Malenka R.C. (2008). Synaptic plasticity: multiple forms, functions, and mechanisms. Neuropsychopharmacology.

[2] Ho V.M., Lee J.-A., Martin K.C. (2011). The cell biology of synaptic plasticity. Science.

[3] Bliss T.V., Lømo T. (1973). Long-lasting potentiation of synaptic transmission in the dentate area of the anaesthetized rabbit following stimulation of the perforant path. J. Physiol..

[4] Lomo T. (1966). Acta Physiologica Scandinavica.

[5] Collingridge G.L., Volianskis A., Bannister N., France G., Hanna L., Mercier M., Tidball P., Fang G., Irvine M.W., Costa B.M. (2013). The nmda receptor as a target for cognitive enhancement. Neuropharmacology.

[6] Wang J., Chai A., Zhou Q., Lv L., Wang L., Yang Y., Xu L. (2013). Chronic clomipramine treatment reverses core symptom of depression in subordinate tree shrews. PLoS One.

[7] Hansen N., Manahan-Vaughan D. (2015). Hippocampal long-term potentiation that is elicited by perforant path stimulation or that occurs in conjunction with spatial learning is tightly controlled by beta-adrenoreceptors and the locus coeruleus. Hippocampus.

[8] Ito W., Fusco B., Morozov A. (2020). Disinhibition-assisted long-term potentiation in the prefrontal-amygdala pathway via suppression of somatostatin-expressing interneurons. Neurophotonics.

[9] Sakurai M. (1987). Synaptic modification of parallel fibre-purkinje cell transmission in in vitro guinea-pig cerebellar slices. J. Physiol..

[10] Hirano T. (1990). Depression and potentiation of the synaptic transmission between a granule cell and a purkinje cell in rat cerebellar culture. Neurosci. Lett..

[11] Huang Y.Y., Kandel E.R. (2005). Theta frequency stimulation induces a local form of late phase ltp in the ca1 region of the hippocampus. Learn. Mem..

[12] Pang K.K.L., Sharma M., Krishna-K K., Behnisch T., Sajikumar S. (2019). Long-term population spike-timing-dependent plasticity promotes synaptic tagging but not cross-tagging in rat hippocampal area ca1. Proc. Natl. Acad. Sci. USA.

[13] Guzman E., Cheng Z., Hansma P.K., Tovar K.R., Petzold L.R., Kosik K.S. (2021). Extracellular detection of neuronal coupling. Sci. Rep..

[14] Anisimova M., van Bommel B., Wang R., Mikhaylova M., Wiegert J.S., Oertner T.G., Gee C.E. (2022). Spike-timing-dependent plasticity rewards synchrony rather than causality. Cereb. Cortex.

[15] Meijer M., Rehbach K., Brunner J.W., Classen J.A., Lammertse H.C.A., van Linge L.A., Schut D., Krutenko T., Hebisch M., Cornelisse L.N. (2019). A single-cell model for synaptic transmission and plasticity in human ipsc-derived neurons. Cell Rep..

[16] Chang C.-L., Trimbuch T., Chao H.-T., Jordan J.-C., Herman M.A., Rosenmund C. (2014). Investigation of synapse formation and function in a glutamatergic-gabaergic two-neuron microcircuit. J. Neurosci..

[17] Taylor A.M., Blurton-Jones M., Rhee S.W., Cribbs D.H., Cotman C.W., Jeon N.L. (2005). A microfluidic culture platform for cns axonal injury, regeneration and transport. Nat. Methods.

[18] Forró C., Thompson-Steckel G., Weaver S., Weydert S., Ihle S., Dermutz H., Aebersold M.J., Pilz R., Demkó L., Vörös J. (2018). Modular microstructure design to build neuronal networks of defined functional connectivity. Biosens. Bioelectron..

[19] Moutaux E., Charlot B., Genoux A., Saudou F., Cazorla M. (2018). An integrated microfluidic/microelectrode array for the study of activity-dependent intracellular dynamics in neuronal networks. Lab Chip.

[20] Gupta P., Shinde A., Illath K., Kar S., Nagai M., Tseng F.-G., Santra T.S. (2022). Microfluidic platforms for single neuron analysis. Mater. Today Bio.

[21] Villard C. (2023). Spatial confinement: A spur for axonal growth. Semin. Cell Dev. Biol..

[22] Vulić K., Amos G., Ruff T., Kasm R., Ihle S.J., Küchler J., Vörös J., Weaver S. (2024). Impact of microchannel width on axons for brain-on-chip applications. Lab Chip.

[23] Zhang H., Wang P., Huang N., Zhao L., Su Y., Li L., Bian S., Sawan M. (2023). Single neurons on microelectrode array chip: manipulation and analyses. Front. Bioeng. Biotechnol..

[24] Taylor A.M., Dieterich D.C., Ito H.T., Kim S.A., Schuman E.M. (2010). Microfluidic local perfusion chambers for the visualization and manipulation of synapses. Neuron.

[25] Mateus J.C., Weaver S., Van Swaay D., Renz A.F., Hengsteler J., Aguiar P., Vörös J. (2022). Nanoscale Patterning of in Vitro Neuronal Circuits. ACS Nano.

[26] Duru J., Küchler J., Ihle S.J., Forró C., Bernardi A., Girardin S., Hengsteler J., Wheeler S., Vörös J., Ruff T. (2022). Engineered biological neural networks on high density cmos microelectrode arrays. Front. Neurosci..

[27] Girardin S., Ihle S.J., Menghini A., Krubner M., Tognola L., Duru J., Fruh I., Müller M., Ruff T., Vörös J. (2023). Engineering circuits of human ipsc-derived neurons and rat primary glia. Front. Neurosci..

[28] Müller J., Ballini M., Livi P., Chen Y., Radivojevic M., Shadmani A., Viswam V., Jones I.L., Fiscella M., Diggelmann R. (2015). High-resolution cmos mea platform to study neurons at subcellular, cellular, and network levels. Lab Chip.

[29] Hecht-Nielsen R., McKenna T. (2003). Computational Models for Neuroscience: Human Cortical Information Processing.

[30] Bourjaily M.A., Miller P. (2011). Synaptic plasticity and connectivity requirements to produce stimulus-pair specific responses in recurrent networks of spiking neurons. PLoS Comput. Biol..

[31] Gerstner W., Kempter R., van Hemmen J.L., Wagner H. (1996). A neuronal learning rule for sub-millisecond temporal coding. Nature.

[32] Markram H., Sakmann B. (1995).

[33] Rossum M., Bi G., Turrigiano G. (2000). Stable hebbian learning from spike timing-dependent plasticity. J. Neurosci..

[34] Arendt K.L., Sarti F., Chen L. (2013). Chronic inactivation of a neural circuit enhances ltp by inducing silent synapse formation. J. Neurosci..

[35] Welzel O., Tischbirek C.H., Jung J., Kohler E.M., Svetlitchny A., Henkel A.W., Kornhuber J., Groemer T.W. (2010). Synapse clusters are preferentially formed by synapses with large recycling pool sizes. PLoS One.

[36] Honnuraiah S., Narayanan R. (2013). A calcium-dependent plasticity rule for hcn channels maintains activity homeostasis and stable synaptic learning. PLoS One.

[37] Linderman S.W., Gershman S.J. (2017). Using computational theory to constrain statistical models of neural data. Curr. Opin. Neurobiol..

[38] Geminiani A., Casellato C., Locatelli F., Prestori F., Pedrocchi A., D’Angelo E. (2018). Complex dynamics in simplified neuronal models: Reproducing golgi cell electroresponsiveness. Front. Neuroinform..

[39] Wen J., Peitz M., Brüstle O. (2022). A defined human-specific platform for modeling neuronal network stimulation in vitro and in silico. J. Neurosci. Methods.

[40] Doorn N., van Hugte E.J.H., Ciptasari U., Mordelt A., Meijer H.G.E., Schubert D., Frega M., Nadif Kasri N., van Putten M.J.A.M. (2023). An in silico and in vitro human neuronal network model reveals cellular mechanisms beyond nav1.1 underlying dravet syndrome. Stem Cell Rep..

[41] Bang S., Lee B.-J., Lee S.-R., Na S., Jang J.M., Kang M., Kim S.-Y., Min D.-H., Song J.M., Ho W.-K., Jeon N.L. (2018). Reliable autapse formation using the single-cell patterning method. Biofabrication.

[42] Rhee H.J., Shaib A.H., Rehbach K., Lee C., Seif P., Thomas C., Gideons E., Guenther A., Krutenko T., Hebisch M. (2019). An autaptic culture system for standardized analyses of ipsc-derived human neurons. Cell Rep..

[43] Jäckel D., Bakkum D.J., Russell T.L., Müller J., Radivojevic M., Frey U., Franke F., Hierlemann A. (2017). Combination of high-density microelectrode array and patch clamp recordings to enable studies of multisynaptic integration. Sci. Rep..

[44] Cooper L.N., Bear M.F. (2012). The bcm theory of synapse modification at 30: interaction of theory with experiment. Nat. Rev. Neurosci..

[45] Lenk K., Priwitzer B., Ylä-Outinen L., Tietz L.H.B., Narkilahti S., Hyttinen J.A.K. (2016). Simulation of developing human neuronal cell networks. Biomed. Eng. Online.

[46] Wagenaar D.A., Pine J., Potter S.M. (2006). Searching for plasticity in dissociated cortical cultures on multi-electrode arrays. J. Negat. Results Biomed..

[47] Hodgkin A.L., Huxley A.F. (1952). A quantitative description of membrane current and its application to conduction and excitation in nerve. J. Physiol..

[48] Papamakarios G., Murray I., Lee D., Sugiyama M., Luxburg U., Guyon I., Garnett R. (2016). https://proceedings.neurips.cc/paper_files/paper/2016/file/6aca97005c68f1206823815f66102863-Paper.pdf.

[49] Cranmer K., Brehmer J., Louppe G. (2020). The frontier of simulation-based inference. Proc. Natl. Acad. Sci. USA.

[50] Amos G., Ihle S.J., Clément B.F., Duru J., Girardin S., Maurer B., Delipinar T., Vörös J., Ruff T. (2024). Engineering an in vitro retinothalamic nerve model. Front. Neurosci..

[51] Clément B.F., Petrella L., Wallimann L., Duru J., Tringides C.M., Vörös J., Ruff T. (2025). An in vitro platform for characterizing axonal electrophysiology of individual human ipsc-derived nociceptors. Biosens. Bioelectron..

[52] Girardin S., Clément B., Ihle S.J., Weaver S., Petr J.B., Mateus J.C., Duru J., Krubner M., Forró C., Ruff T. (2022). Topologically controlled circuits of human ipsc-derived neurons for electrophysiology recordings. Lab Chip.

[53] Renault R., Durand J.-B., Viovy J.-L., Villard C. (2016). Asymmetric axonal edge guidance: a new paradigm for building oriented neuronal networks. Lab Chip.

[54] Holloway P.M., Hallinan G.I., Hegde M., Lane S.I.R., Deinhardt K., West J. (2019). Asymmetric confinement for defining outgrowth directionality. Lab Chip.

[55] Winter-Hjelm N., Tomren B., Sikorski P., Sandvig A., Sandvig I. (2023). Structure-function dynamics of engineered, modular neuronal networks with controllable afferent-efferent connectivity. J. Neural. Eng..

[56] Ming Y., Abedin M.J., Tatic-Lucic S., Berdichevsky Y. (2021). Microdevice for directional axodendritic connectivity between micro 3d neuronal cultures. Microsyst. Nanoeng..

[57] Pan L., Alagapan S., Franca E., Leondopulos S.S., DeMarse T.B., Brewer G.J., Wheeler B.C. (2015). An in vitro method to manipulate the direction and functional strength between neural populations. Front. Neural Circuits.

[58] Lam R.S., Töpfer F.M., Wood P.G., Busskamp V., Bamberg E. (2017). Functional maturation of human stem cell-derived neurons in long-term cultures. PLoS One.

[59] Duru J., Maurer B., Giles Doran C., Jelitto R., Küchler J., Ihle S.J., Ruff T., John R., Genocchi B., Vörös J. (2023). Investigation of the input-output relationship of engineered neural networks using high-density microelectrode arrays. Biosens. Bioelectron..

[60] Hoffmeister B., Jänig W., Lisney S.J. (1991). A proposed relationship between circumference and conduction velocity of unmyelinated axons from normal and regenerated cat hindlimb cutaneous nerves. Neuroscience.

[61] Horowitz A., Barazany D., Tavor I., Bernstein M., Yovel G., Assaf Y. (2015). In vivo correlation between axon diameter and conduction velocity in the human brain. Brain Struct. Funct..

[62] Odawara A., Katoh H., Matsuda N., Suzuki I. (2016). Physiological maturation and drug responses of human induced pluripotent stem cell-derived cortical neuronal networks in long-term culture. Sci. Rep..

[63] Zhang Y., Pak C., Han Y., Ahlenius H., Zhang Z., Chanda S., Marro S., Patzke C., Acuna C., Covy J. (2013). Rapid single-step induction of functional neurons from human pluripotent stem cells. Neuron.

[64] Debanne D., Gähwiler B.H., Thompson S.M. (1996). Cooperative interactions in the induction of long-term potentiation and depression of synaptic excitation between hippocampal ca3-ca1 cell pairs in vitro. Proc. Natl. Acad. Sci. USA.

[65] Wittenberg G.M., Wang S.S.-H. (2006). Malleability of spike-timing-dependent plasticity at the ca3-ca1 synapse. J. Neurosci..

[66] Schreiber T. (2000). Measuring information transfer. Phys. Rev. Lett..

[67] Paluš M., Komárek V., Hrnčíř Z., Štěrbová K. (2001). Synchronization as adjustment of information rates: Detection from bivariate time series. Phys. Rev..

[68] Wollstadt P., Martínez-Zarzuela M., Vicente R., Pernas F.J.D., Wibral M. (2014). Efficient transfer entropy analysis of non-stationary neural time series. PLoS One.

[69] Novelli L., Wollstadt P., Mediano P., Wibral M., Lizier J.T. (2019). Large-scale directed network inference with multivariate transfer entropy and hierarchical statistical testing. Netw. Neurosci..

[70] Janzing, D., Balduzzi, D., Grosse-Wentrup, M., and Schölkopf, B. (2013). Quantifying causal influences.

[71] Bullmann T., Kaas T., Ritzau-Jost A., Wöhner A., Kirmann T., Rizalar F.S., Holzer M., Nerlich J., Puchkov D., Geis C. (2024). Human ipsc-derived neurons with reliable synapses and large presynaptic action potentials. J. Neurosci..

[72] Morfini G.A., Bosco D.A., Brown H., Gatto R., Kamińska A., Song Y., Molla L., Baker L., Marangoni M.N., Berth S. (2013). Inhibition of fast axonal transport by pathogenic sod1 involves activation of p38 map kinase. PLoS One.

[73] Kanaan N.M., Pigino G.F., Brady S.T., Lazarov O., Binder L.I., Morfini G.A. (2013). Axonal degeneration in alzheimer’s disease: when signaling abnormalities meet the axonal transport system. Exp. Neurol..

[74] Williams S.R., Stuart G.J. (2002). Dependence of epsp efficacy on synapse location in neocortical pyramidal neurons. Science.

[75] Hodgkin A.L., Huxley A.F. (1939). Action potentials recorded from inside a nerve fibre. Nature.

[76] Traub R.D., Wong R.K., Miles R., Michelson H. (1991). A model of a ca3 hippocampal pyramidal neuron incorporating voltage-clamp data on intrinsic conductances. J. Neurophysiol..

[77] Gonçalves P.J., Lueckmann J.-M., Deistler M., Nonnenmacher M., Öcal K., Bassetto G., Chintaluri C., Podlaski W.F., Haddad S.A., Vogels T.P. (2020). Training deep neural density estimators to identify mechanistic models of neural dynamics. eLife.

[78] Lueckmann J.-M., Goncalves P.J., Bassetto G., Öcal K., Nonnenmacher M., Macke J.H., Guyon I., Luxburg U.V., Bengio S., Wallach H., Fergus R., Vishwanathan S., Garnett R. (2017). https://proceedings.neurips.cc/paper_files/paper/2017/file/addfa9b7e234254d26e9c7f2af1005cb-Paper.pdf.

[79] Rushton W. (1951). A theory of the effects of fibre size in medullated nerve. J. Physiol..

[80] Kessels H.W., Malinow R. (2009). Synaptic ampa receptor plasticity and behavior. Neuron.

[81] Rao V.R., Finkbeiner S. (2007). Nmda and ampa receptors: old channels, new tricks. Trends Neurosci..

[82] Malenka R.C., Bear M.F. (2004). Ltp and ltd: An embarrassment of riches. Neuron.

[83] Debanne D., Inglebert Y., Russier M. (2019). Plasticity of intrinsic neuronal excitability. Curr. Opin. Neurobiol..

[84] Diering G.H., Huganir R.L. (2018). The ampa receptor code of synaptic plasticity. Neuron.

[85] Suzuki I., Sugio Y., Moriguchi H., Jimbo Y., Yasuda K. (2004). Modification of a neuronal network direction using stepwise photo-thermal etching of an agarose architecture. J. Nanobiotechnol..

[86] Sgro A.E., Nowak A.L., Austin N.S., Custer K.L., Allen P.B., Chiu D.T., Bajjalieh S.M. (2011). A high-throughput method for generating uniform microislands for autaptic neuronal cultures. J. Neurosci. Methods.

[87] Huang W.-H., Cheng W., Zhang Z., Pang D.-W., Wang Z.-L., Cheng J.-K., Cui D.-F. (2004). Transport, location, and quantal release monitoring of single cells on a microfluidic device. Anal. Chem..

[88] Deleglise B., Magnifico S., Duplus E., Vaur P., Soubeyre V., Belle M., Vignes M., Viovy J.L., Brugg B., Jacotot E., Peyrin J.M. (2014). beta-amyloid induces a dying-back process and remote trans-synaptic alterations in a microfluidic-based reconstructed neuronal network. Acta Neuropathol. Commun..

[89] Kagan B.J., Kitchen A.C., Tran N.T., Habibollahi F., Khajehnejad M., Parker B.J., Bhat A., Rollo B., Razi A., Friston K.J. (2022). In vitro neurons learn and exhibit sentience when embodied in a simulated game-world. Neuron.

[90] Osaki T., Duenki T., Chow S.Y.A., Ikegami Y., Beaubois R., Levi T., Nakagawa-Tamagawa N., Hirano Y., Ikeuchi Y. (2024). Complex activity and short-term plasticity of human cerebral organoids reciprocally connected with axons. Nat. Commun..

[91] Chindemi G., Abdellah M., Amsalem O., Benavides-Piccione R., Delattre V., Doron M., Ecker A., Jaquier A.T., King J., Kumbhar P. (2022). A calcium-based plasticity model for predicting long-term potentiation and depression in the neocortex. Nat. Commun..

[92] Pfister J.-P., Gerstner W. (2006). Triplets of spikes in a model of spike timing-dependent plasticity. J. Neurosci..

[93] Bienenstock E.L., Cooper L.N., Munro P.W. (1982). Theory for the development of neuron selectivity: orientation specificity and binocular interaction in visual cortex. J. Neurosci..

[94] Debanne D., Gähwiler B.H., Thompson S.M. (1994). Asynchronous pre- and postsynaptic activity induces associative long-term depression in area ca1 of the rat hippocampus in vitro. Proc. Natl. Acad. Sci. USA.

[95] Markram H., Lübke J., Frotscher M., Sakmann B. (1997). Regulation of synaptic efficacy by coincidence of postsynaptic aps and epsps. Science.

[96] Bi G.-q., Poo M.-m. (1998). Synaptic modifications in cultured hippocampal neurons: Dependence on spike timing, synaptic strength, and postsynaptic cell type. J. Neurosci..

[97] Froemke R.C., Dan Y. (2002). Spike-timing-dependent synaptic modification induced by natural spike trains. Nature.

[98] Agnes E.J., Vogels T.P. (2024). Co-dependent excitatory and inhibitory plasticity accounts for quick, stable and long-lasting memories in biological networks. Nat. Neurosci..

[99] Carvalho T.P., Buonomano D.V. (2009). Differential effects of excitatory and inhibitory plasticity on synaptically driven neuronal input–output functions. Neuron.

[100] Armano S., Rossi P., Taglietti V., D’Angelo E. (2000). Long-term potentiation of intrinsic excitability at the mossy fiber–granule cell synapse of rat cerebellum. J. Neurosci..

[101] Ganguly K., Kiss L., Poo M. (2000). Enhancement of presynaptic neuronal excitability by correlated presynaptic and postsynaptic spiking. Nat. Neurosci..

[102] Li C.y., Lu J.t., Wu C.p., Duan S.m., Poo M.m. (2004). Bidirectional modification of presynaptic neuronal excitability accompanying spike timing-dependent synaptic plasticity. Neuron.

[103] Daoudal G., Hanada Y., Debanne D. (2002). Bidirectional plasticity of excitatory postsynaptic potential (epsp)-spike coupling in ca1 hippocampal pyramidal neurons. Proc. Natl. Acad. Sci. USA.

[104] Wang Z., Xu N.l., Wu C.p., Duan S., Poo M.m. (2003). Bidirectional changes in spatial dendritic integration accompanying long-term synaptic modifications. Neuron.

[105] Xu J., Kang N., Jiang L., Nedergaard M., Kang J. (2005). Activity-dependent long-term potentiation of intrinsic excitability in hippocampal ca1 pyramidal neurons. J. Neurosci..

[106] Campanac E., Debanne D. (2008). Spike timing-dependent plasticity: a learning rule for dendritic integration in rat ca1 pyramidal neurons. J. Physiol..

[107] Giorgetti E., Panesar M., Zhang Y., Joller S., Ronco M., Obrecht M., Lambert C., Accart N., Beckmann N., Doelemeyer A. (2019). Modulation of microglia by voluntary exercise or csf1r inhibition prevents age-related loss of functional motor units. Cell Rep..

[108] Brewer G.J., Torricelli J.R., Evege E.K., Price P.J. (1993). Optimized survival of hippocampal neurons in b27-supplemented neurobasal, a new serum-free medium combination. J. Neurosci. Res..

[109] Schindelin J., Arganda-Carreras I., Frise E., Kaynig V., Longair M., Pietzsch T., Preibisch S., Rueden C., Saalfeld S., Schmid B. (2012). Fiji: an open-source platform for biological-image analysis. Nat. Methods.

[110] Küchler J., Vulić‡ K., Yao H., Valmaggia C., Ihle S.J., Weaver S., Vörös J. (2025). Engineered biological neuronal networks as basic logic operators. Front. Comput. Neurosci..

[111] Buccino A.P., Hurwitz C.L., Garcia S., Magland J., Siegle J.H., Hurwitz R., Hennig M.H. (2020). Spikeinterface, a unified framework for spike sorting. eLife.

[112] Yger P., Spampinato G.L., Esposito E., Lefebvre B., Deny S., Gardella C., Stimberg M., Jetter F., Zeck G., Picaud S. (2018). A spike sorting toolbox for up to thousands of electrodes validated with ground truth recordings in vitro and in vivo. eLife.

[113] Bossomaier T., Barnett L., Harré M., Lizier J.T., Bossomaier T., Barnett L., Harré M., Lizier J.T. (2016).

[114] Wollstadt P., Lizier J.T., Vicente R., Finn C., Martinez-Zarzuela M., Mediano P., Novelli L., Wibral M. (2019). IDTxl: The Information Dynamics Toolkit xl: a Python package for the efficient analysis of multivariate information dynamics in networks. Journal of Open Source Software.

[115] Varley T.F., Havert D., Fosque L., Alipour A., Weerawongphrom N., Naganobori H., O’Shea L., Pope M., Beggs J. (2024). The serotonergic psychedelic n, n-dipropyltryptamine alters information-processing dynamics in in vitro cortical neural circuits. Netw. Neurosci..

[116] Wibral M., Wollstadt P. (2021). Runtimes and benchmarking pwollstadt/idtxl wiki. https://github.com/pwollstadt/IDTxl/wiki/Runtimes-and-Benchmarking.

[117] Lizier J.T. (2014). Jidt: An information-theoretic toolkit for studying the dynamics of complex systems. Front. Robot. AI.

[118] Haynes W. (2013).

[119] Ito S., Hansen M.E., Heiland R., Lumsdaine A., Litke A.M., Beggs J.M. (2011). Extending transfer entropy improves identification of effective connectivity in a spiking cortical network model. PLoS One.

[120] Nigam S., Shimono M., Ito S., Yeh F.-C., Timme N., Myroshnychenko M., Lapish C.C., Tosi Z., Hottowy P., Smith W.C. (2016). Rich-club organization in effective connectivity among cortical neurons. J. Neurosci..

[121] Hagen E., Næss S., Ness T.V., Einevoll G.T. (2018). Multimodal modeling of neural network activity: computing lfp, ecog, eeg, and meg signals with lfpy 2.0. Front. Neuroinform..

[122] Carnevale N.T., Hines M.L. (2006).

[123] Halnes G., Ness T.V., Næss S., Hagen E., Pettersen K.H., Einevoll G.T. (2024).

[124] Hendrickson E.B., Edgerton J.R., Jaeger D. (2011). The capabilities and limitations of conductance-based compartmental neuron models with reduced branched or unbranched morphologies and active dendrites. J. Comput. Neurosci..

[125] Tejero-Cantero A., Boelts J., Deistler M., Lueckmann J.-M., Durkan C., Gonçalves P., Greenberg D., Macke J. (2020). sbi: A toolkit for simulation-based inference. J. Open Source Softw..

[126] Greenberg D., Nonnenmacher M., Macke J. (2019). International conference on machine learning.

