# Supplementary material for: An integrated in vitro platform and biophysical modeling approach for studying synaptic transmission in isolated neuronal pairs
Source: iScience. 2026 Apr 1;29(5):115488. doi: 10.1016/j.isci.2026.115488 (PMC13092622; doi:10.1016/j.isci.2026.115488)
Supplement: Document S1. Figures S1–S17 and Tables S1–S7 [file mmc1.pdf]

**iScience, Volume *29***

## Supplemental information

# An integrated *in vitro* platform and biophysical modeling approach for studying synaptic transmission in isolated neuronal pairs

**Giulia Amos, Vaiva Vasiliauskaitė, Jens Duru, Maria Leonor Azevedo Saramago, Tim Schmid, Alexandre Suter, Ferran Cid Torren, Joël Küchler, Tobias Ruff, János Vörös, and Katarina Vulić**

# Supplementary Material

## Supplementary Figures

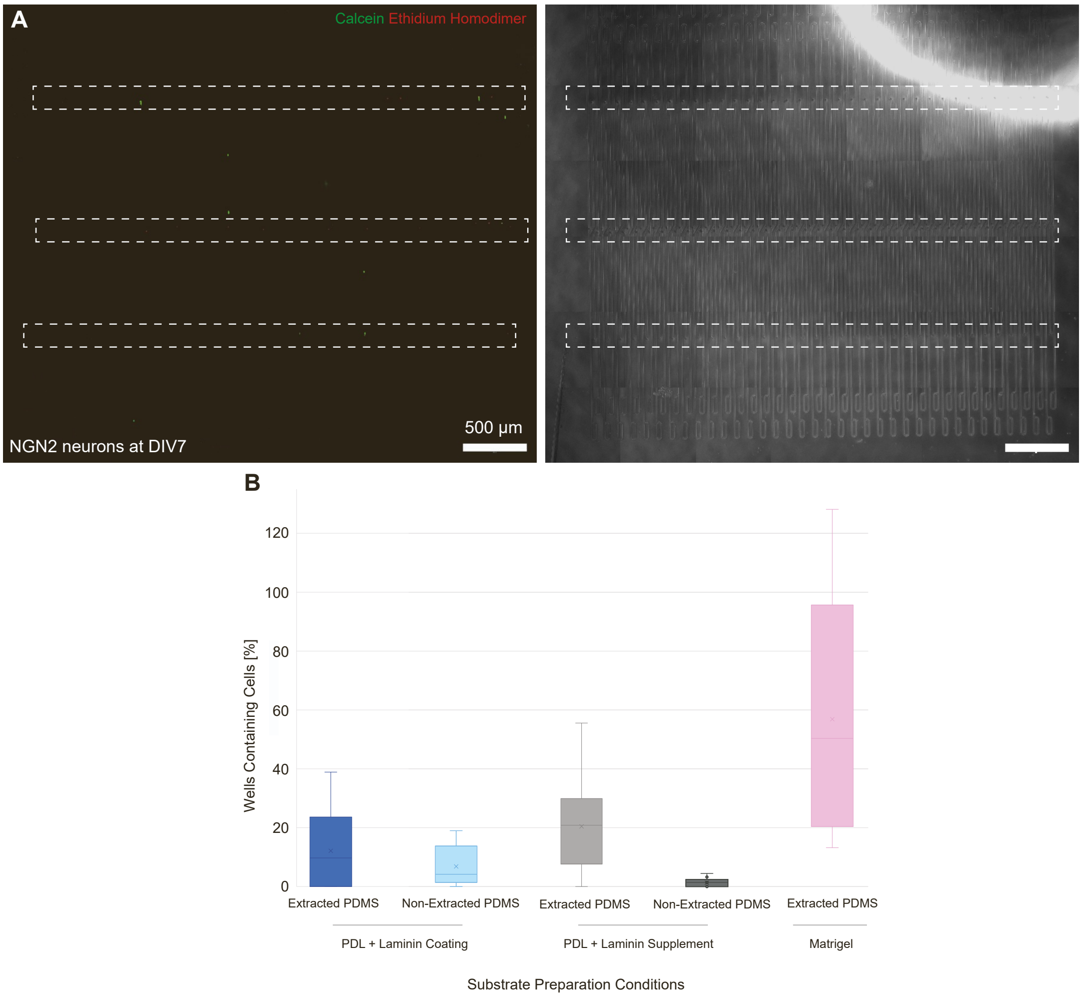


**Figure S1:** Cell culturing condition optimization. **A**) Microscopy image showing low number of isolated cells on DIV7 inside a microstructure placed on the substrate coated with PDL and laminin. The culture was stained with live/dead staining kit consisting of calcein and ethidium homodimer, which is shown on the left. The phase contrast image showing microchannel outline is shown on the right. Well area is highlighted with white dashed squares. **B**) Comparisons of cell abundance inside the microstructures for different substrate preparations. Following cell-adhesive coating conditions are compared: PDL coating and laminin added as a coating before microstructure mounting, PDL coating and laminin added as a medium supplement after microstructure mounting and matrigel as a coating added after microstructure mounting. Extracted and non-extracted PDMS microstructures are compared for the first two conditions. Data are represented as mean ± 95% confidence interval.

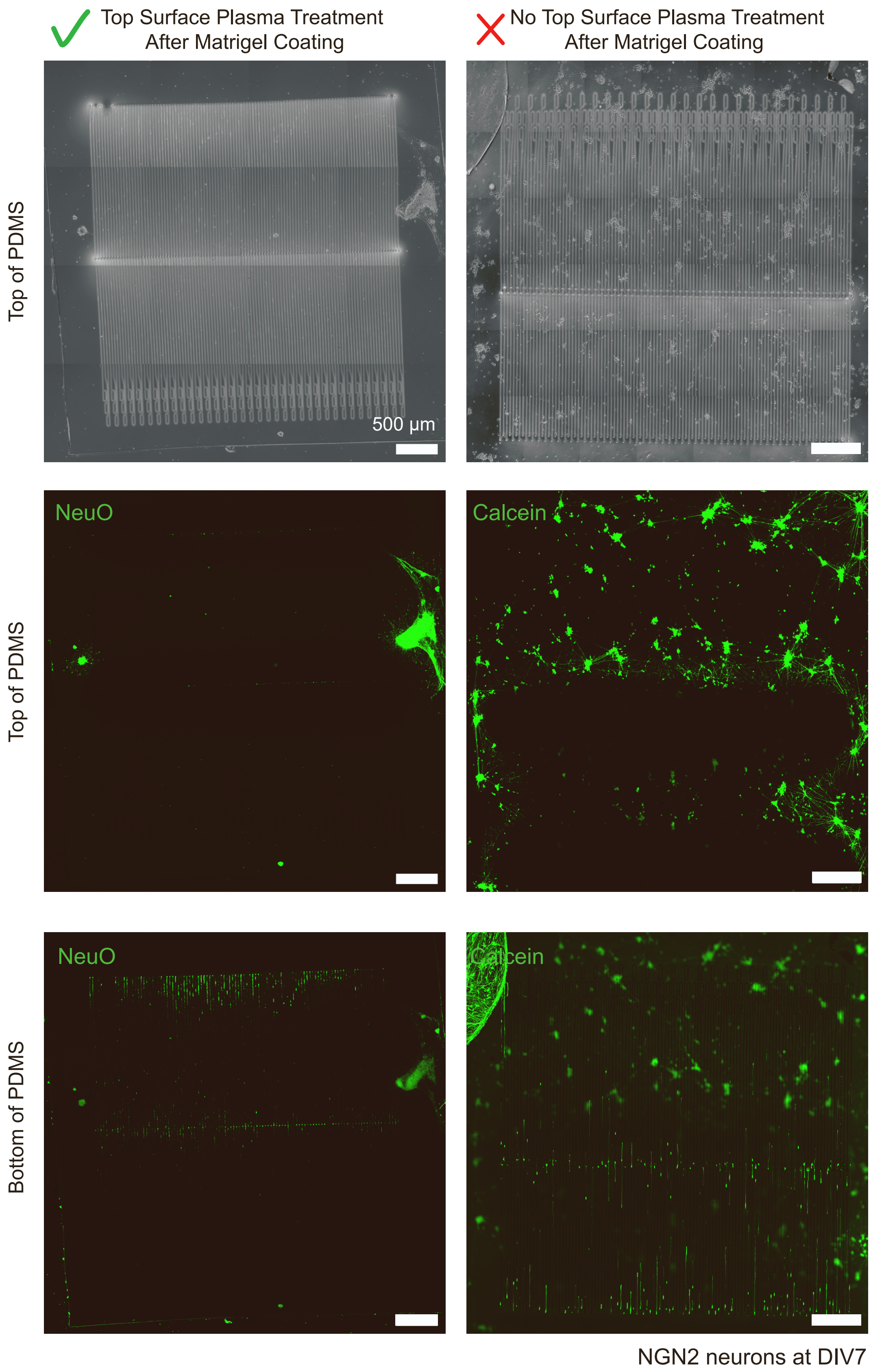


**Figure S2:** Cell overgrowth prevention. Phase contrast (top raw) and fluorescent microscopy images showing the comparison of the PDMS top surface when treating the surface with plasma after matrigel coating (top left, middle left) and when no treatment was applied upon coating (top right, middle right). The abundance of cells inside the microchannels is comparable in both cases (bottom left and right), indicating that matrigel coating inside the microchannels is preserved upon plasma treatment. All images were obtained on DIV 7.

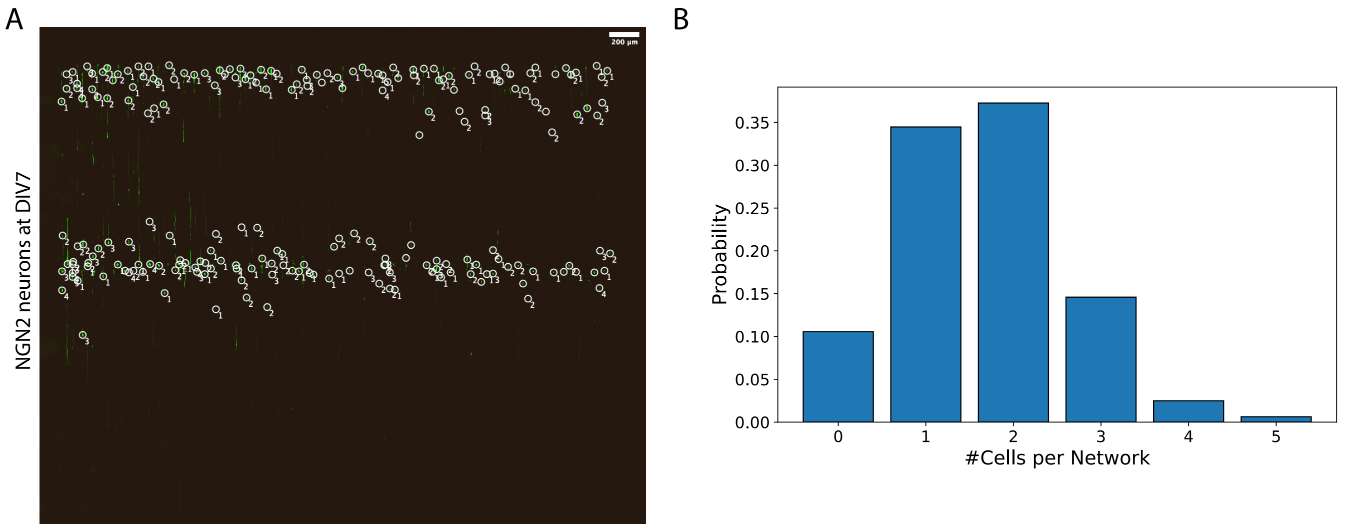


**Figure S3:** Quantification of cell counts per network. **A**) Fluorescence microscopy image of a microstructure containing multiple cells. Individual somata were manually identified and annotated; somata located within the same network were grouped to obtain the cell count per network. **B**) Distribution of the number of cells per network across all analyzed networks (N=322).

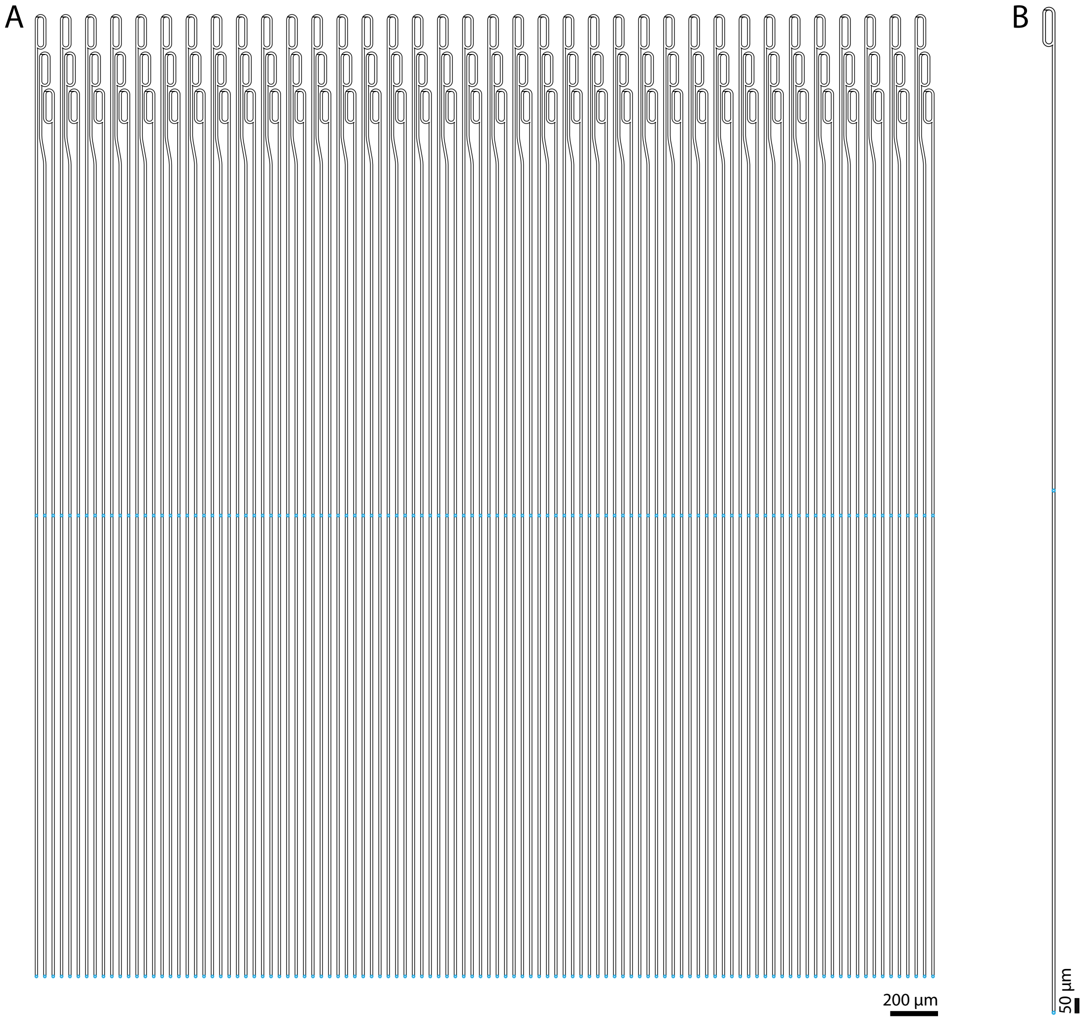


**Figure S4:** Overview of a straight microstructure design. **A**) One microstructure that fits on a HD-CMOS MEA contains 108 isolated microchannels with two rows of 10 µm openings (light blue dots). **B**) Single microchannel example.

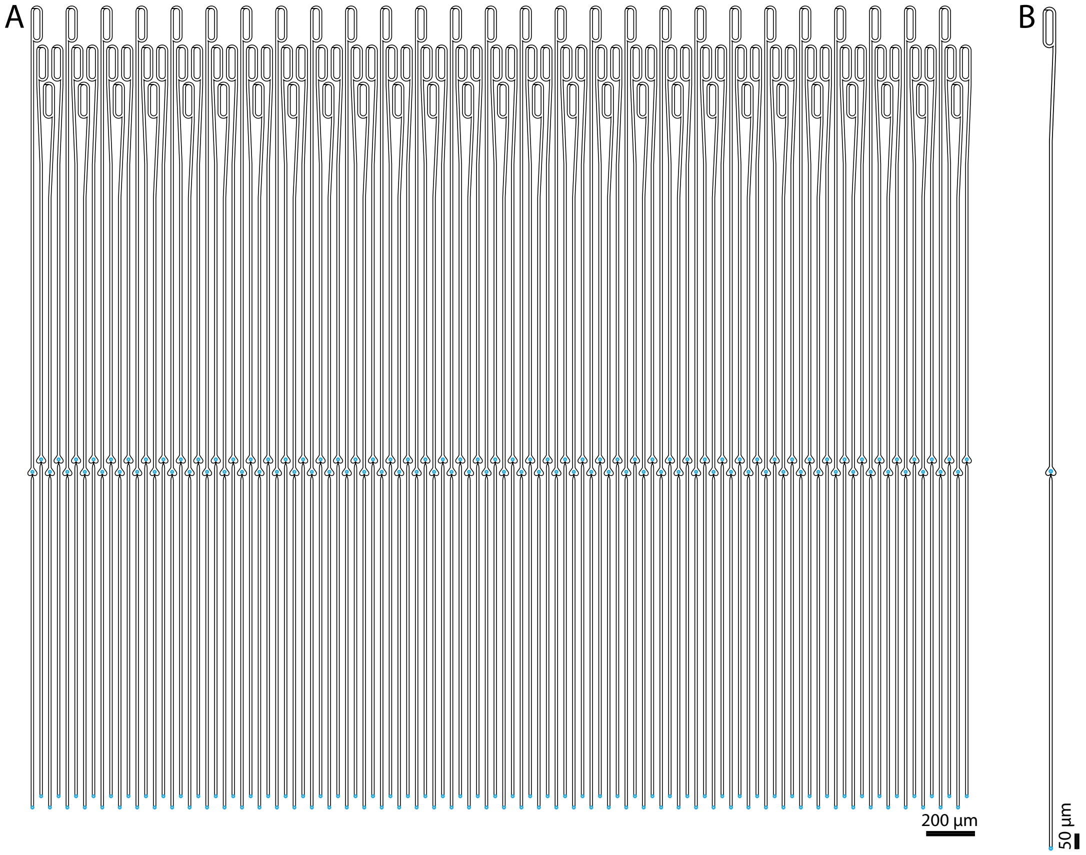


**Figure S5:** Overview of a heart microstructure design. **A**) One microstructure that fits on a HD-CMOS MEA contains 108 isolated microchannels with two rows of 10 μm openings (light blue dots). **B**) Single microchannel example. The heart shape promotes preferential axon growth towards its apex.

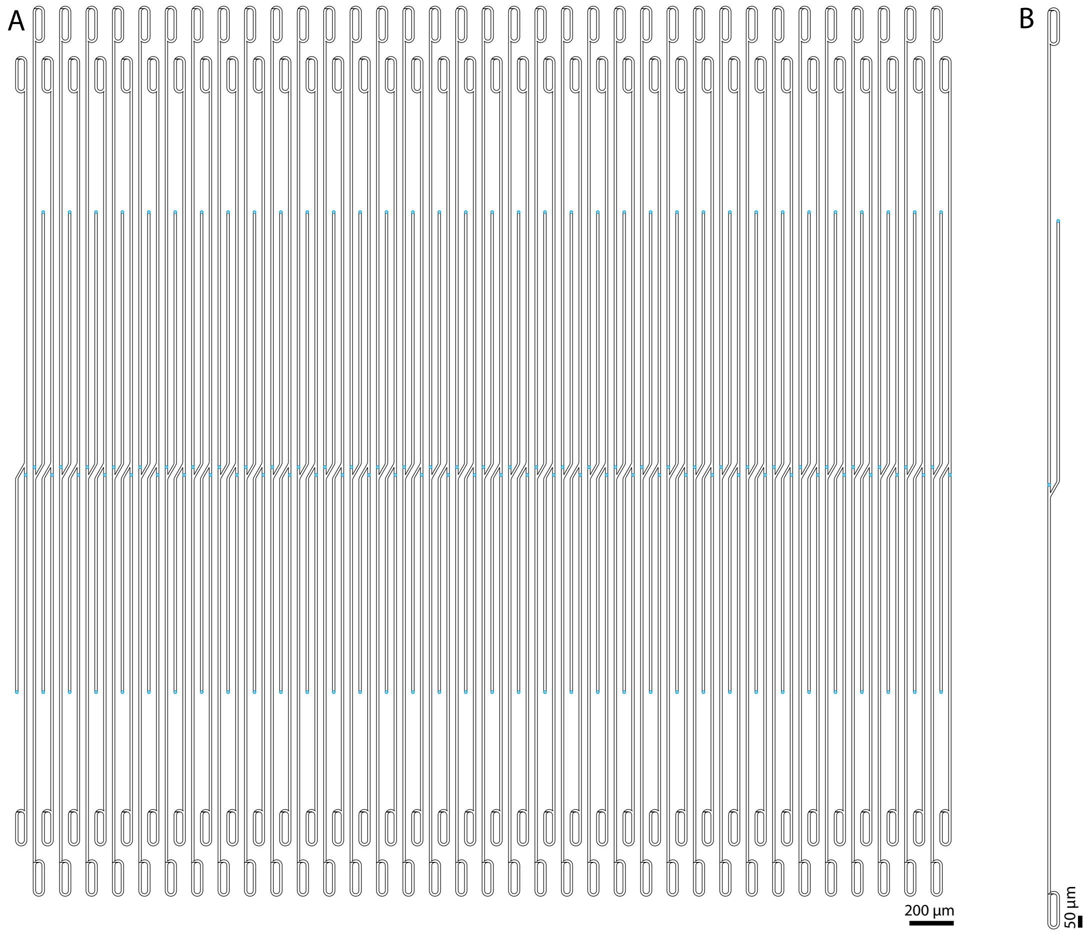


**Figure S6:** Overview of an en-passant microstructure design. **A**) One microstructure that fits on a HD-CMOS MEA contains 72 isolated microchannels with three rows of 10 µm openings (light blue dots). **B**) Single microchannel example. This geometry assures that the axon of the presynaptic neuron grows close enough to connect to the dendrites of the postsynaptic cell.

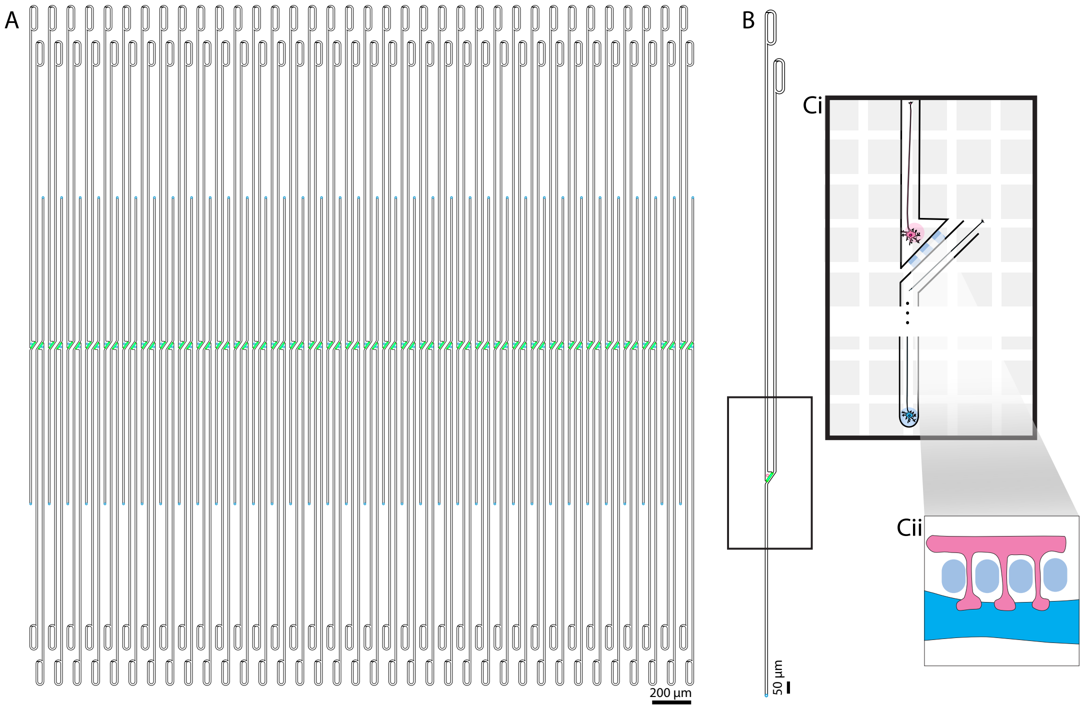


**Figure S7:** Overview of an 1-in-1-out nanochannel microstructure design. Here, nanochannels prevent axons to grow through but enable connection through synaptic spines. **A**) One microstructure that fits on a HD-CMOS MEA contains 72 isolated microchannels with three rows of 10 µm openings (light blue dots). **B**) Single microchannel example.

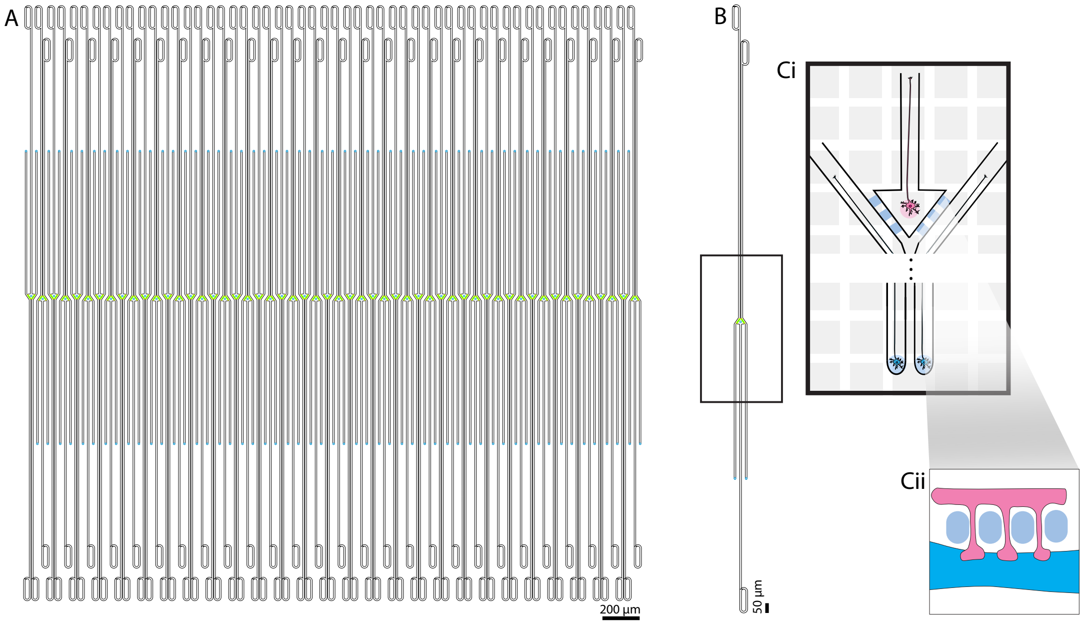


**Figure S8:** Overview of a 2-in-1-out nanochannel microstructure design. **A**) One microstructure that fits on a HD-CMOS MEA contains 44 isolated microchannels with three rows of 10 µm openings (green dots indicating the position of the postsynaptic, light blue dots the presynaptic neurons). **B**) Single microchannel example. The design enables connecting two axons to the dendrites of a postsynaptic neuron.

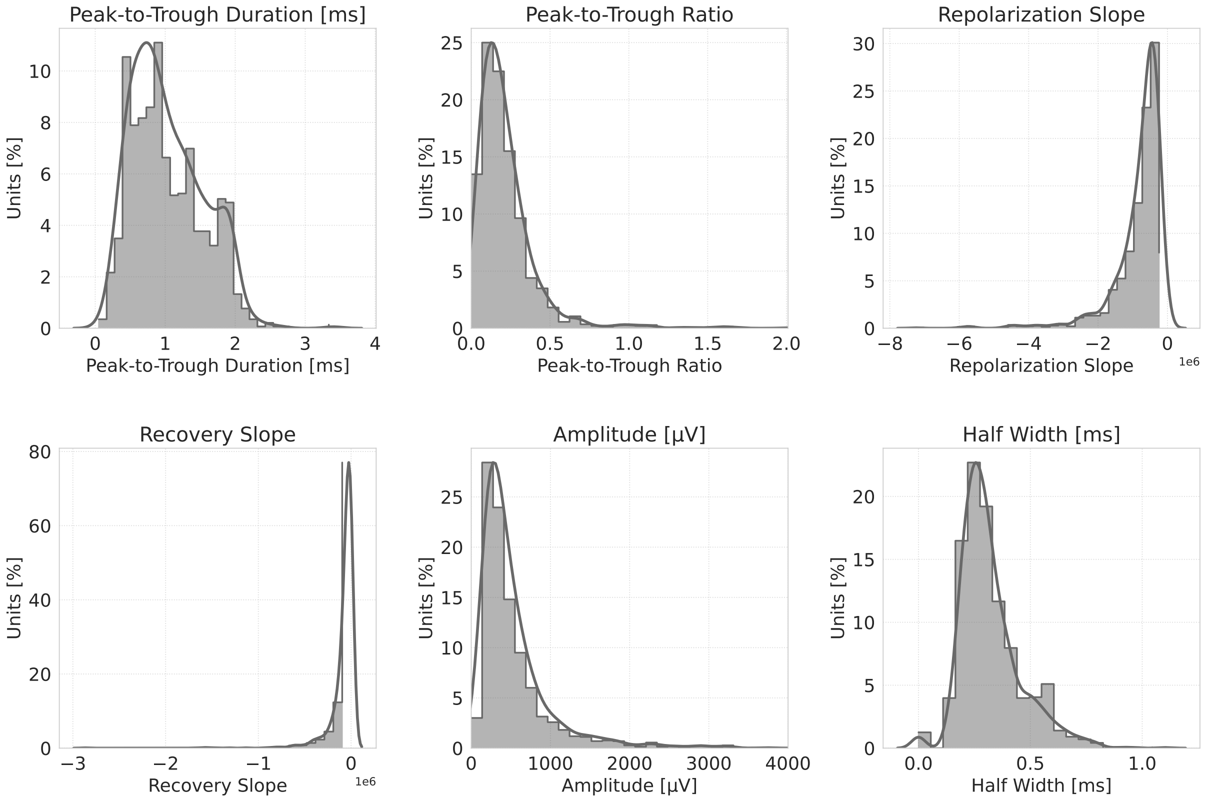


**Figure S9:** Waveform metrics distribution. Waveform metrics extracted by custom-designed data analysis pipeline after the spike sorting performed using the Spikeinterface framework. Histograms show the distribution of values as percentage of total units, with an overlaid kernel density estimate (KDE) curve.

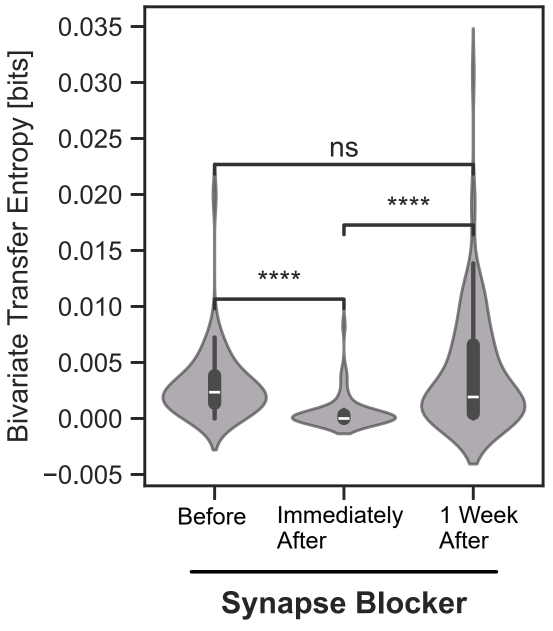


**Figure S10:** Bivariate transfer entropy changes upon synapse blocking. Bivariate transfer entropy calculated for recordings before, immediately after and one week after adding synaptic receptor antagonists (****p $<$ 0.0001, Kruskal-Wallis omnibus tests followed by Dunn's tests, for more details check Table S2). Violin plots show the distribution of data with median and interquartile range.

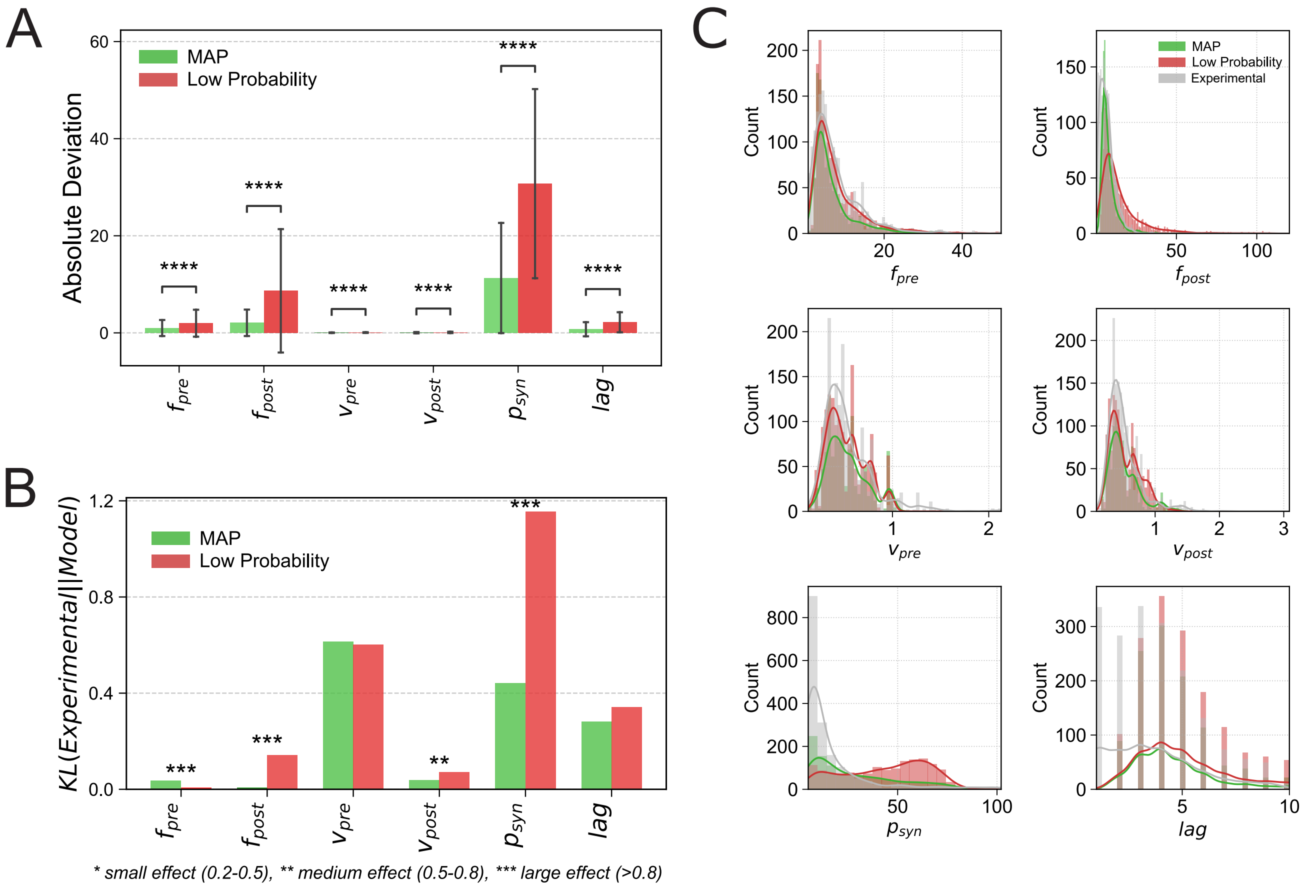


**Figure S11:** High-probability vs. low-probability parameters. **A**) Absolute deviation between parameters sampled from high-probability (maximum a posteriori) and low-probability regions of the distribution (****p $<$ 0.0001, Mann-Whitney-Wilcoxon test). **B**) Kullback-Leibler divergence comparison between maximum a posteriori and low-probability parameter sets. **C**) Visual comparison of maximum a posteriori, low-probability, and experimentally observed parameters.

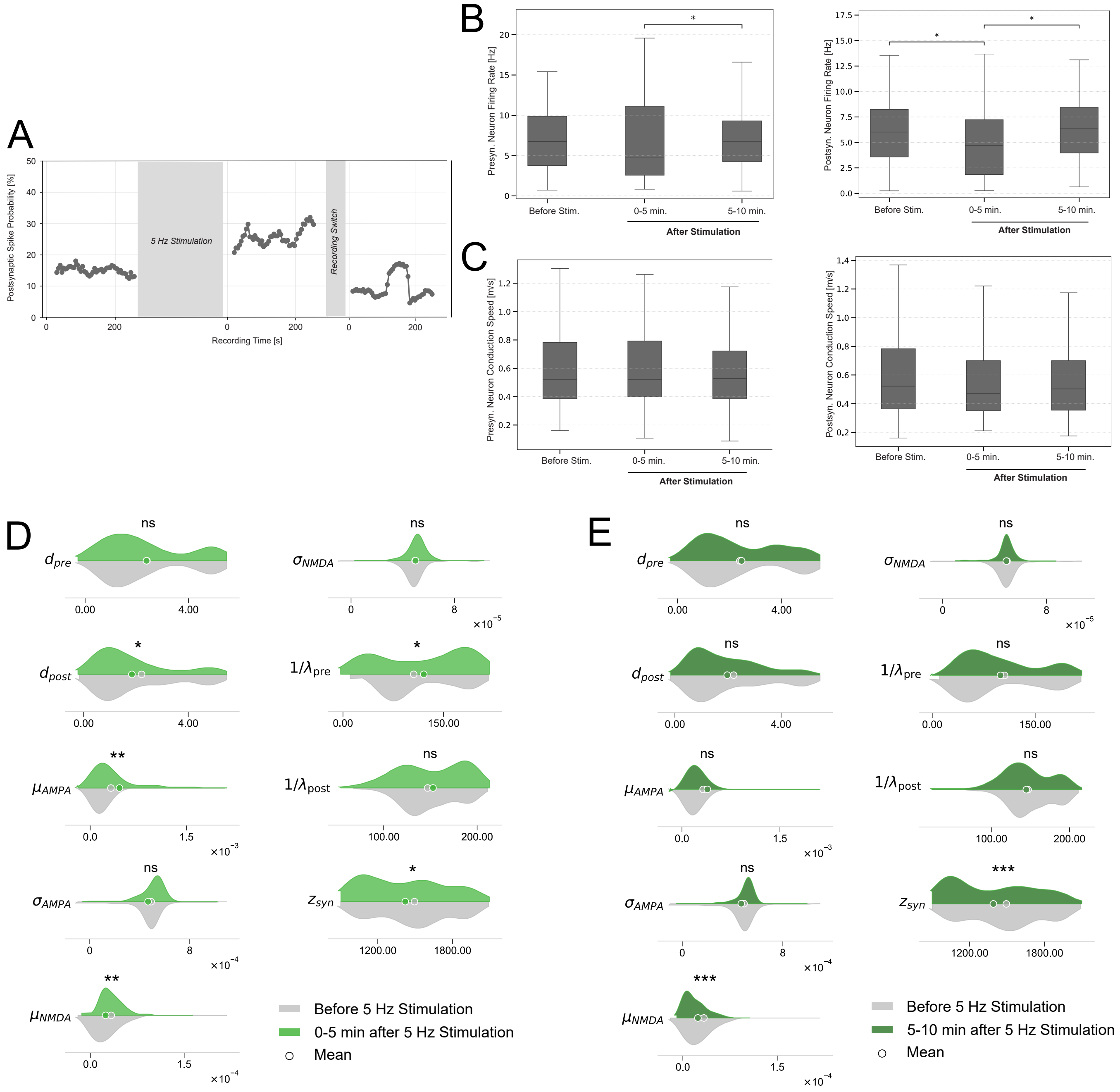


**Figure S12:** Overview of the synaptic and firing parameters in the perturbation experiment. **A**) Mean postsynaptic spike probability over time with corresponding confidence intervals, concatenated across two post-stimulation recording sessions. **B**) Firing rate per neuron extracted from recordings before, immediately after and five minutes after the stimulation protocol was applied. Left plot captures presynaptic neurons (sources), and right plot captures postsynaptic neurons (targets) (*$p < 0.05$, Kruskal-Wallis omnibus tests followed by Dunn's tests). **C**) Conduction speed per neuron extracted from recordings before, immediately after and five minutes after the stimulation protocol was applied. Left plot captures presynaptic neurons (sources), and right plot captures postsynaptic neurons (targets). **D**) Distribution comparison for parameters inferred by the model from recordings before and immediately after stimulation. Significantly different distributions are highlighted in red. **E**) Distribution comparison for parameters inferred by the model from recordings before and five minutes after stimulation. Significantly different distributions are highlighted in red. Box plots in B) and C) show median, interquartile range, and 1.5× IQR whiskers (*$p < 0.05$, **$p < 0.01$, KS-Test).

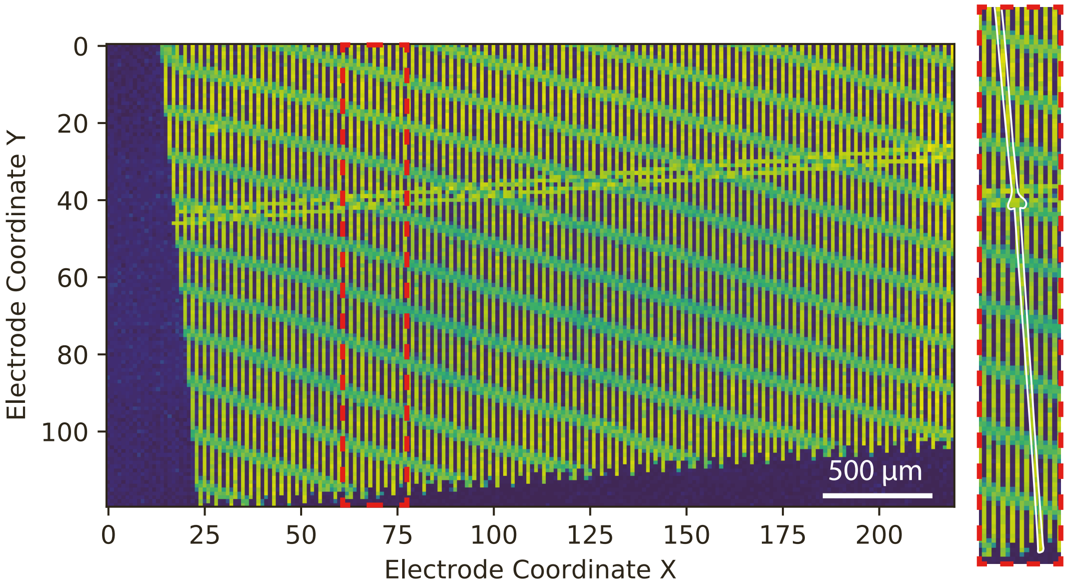


**Figure S13:** Voltage map obtained after microstructure adhesion to the chip. The difference in impedance between electrodes that are covered in PDMS and that are exposed to the liquid enables us to identify the independent networks.

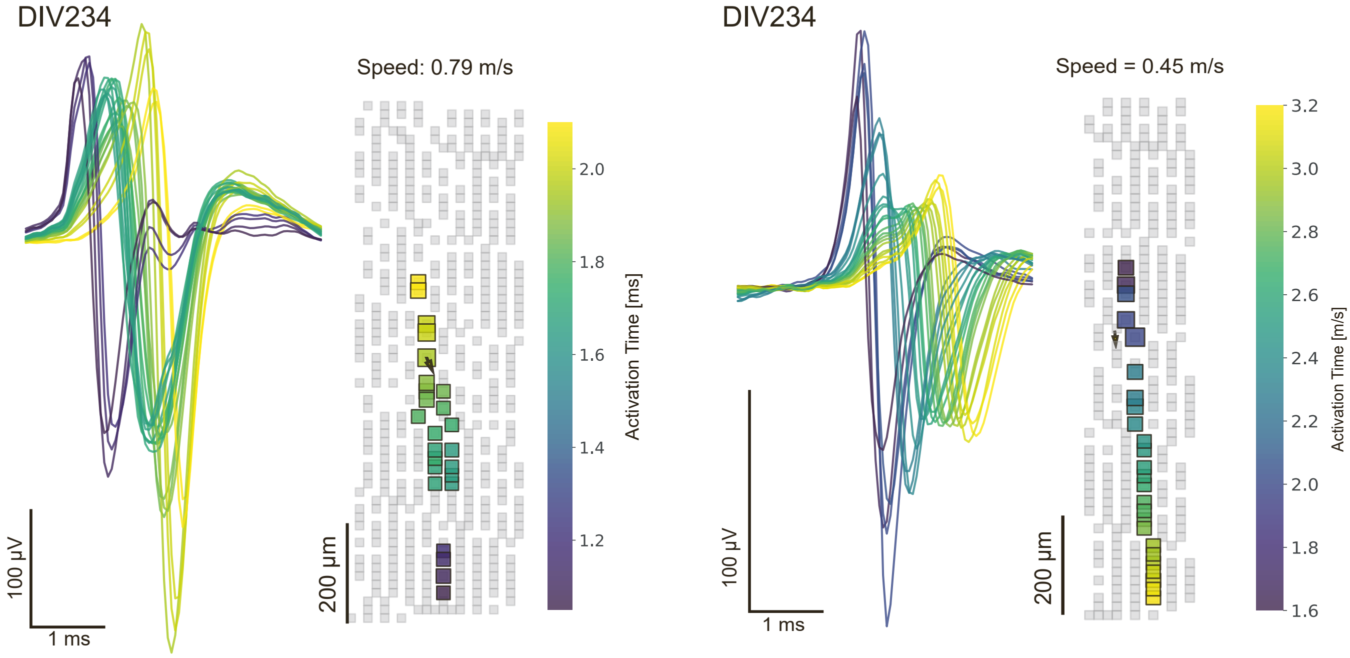


**Figure S14:** Aged culture waveforms. Waveforms extracted from DIV 234 recordings from NGN2 neurons isolated in microchannels.

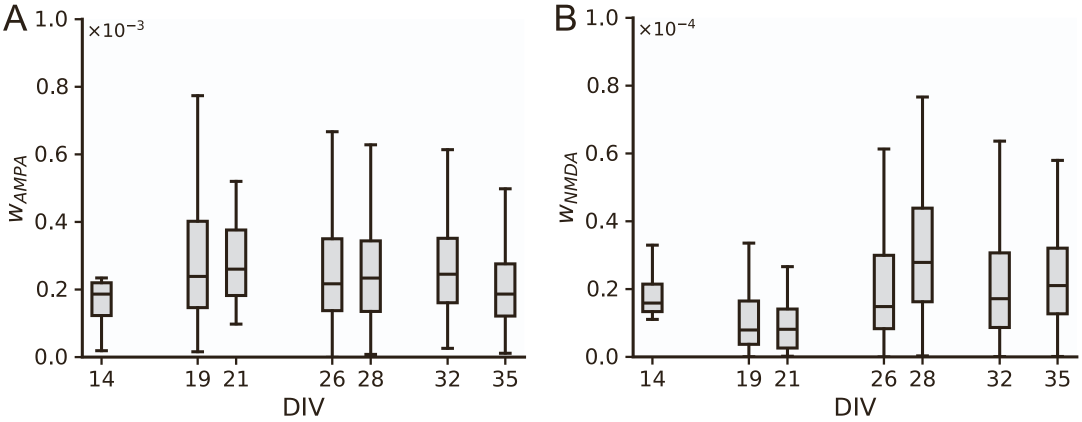


**Figure S15:** Synaptic parameters progression. Progression of mean values of **A)** AMPA and **B)** NMDA receptors, over day *in vitro*, derived using our model. Box plots show median, interquartile range, and 1.5× IQR whiskers.

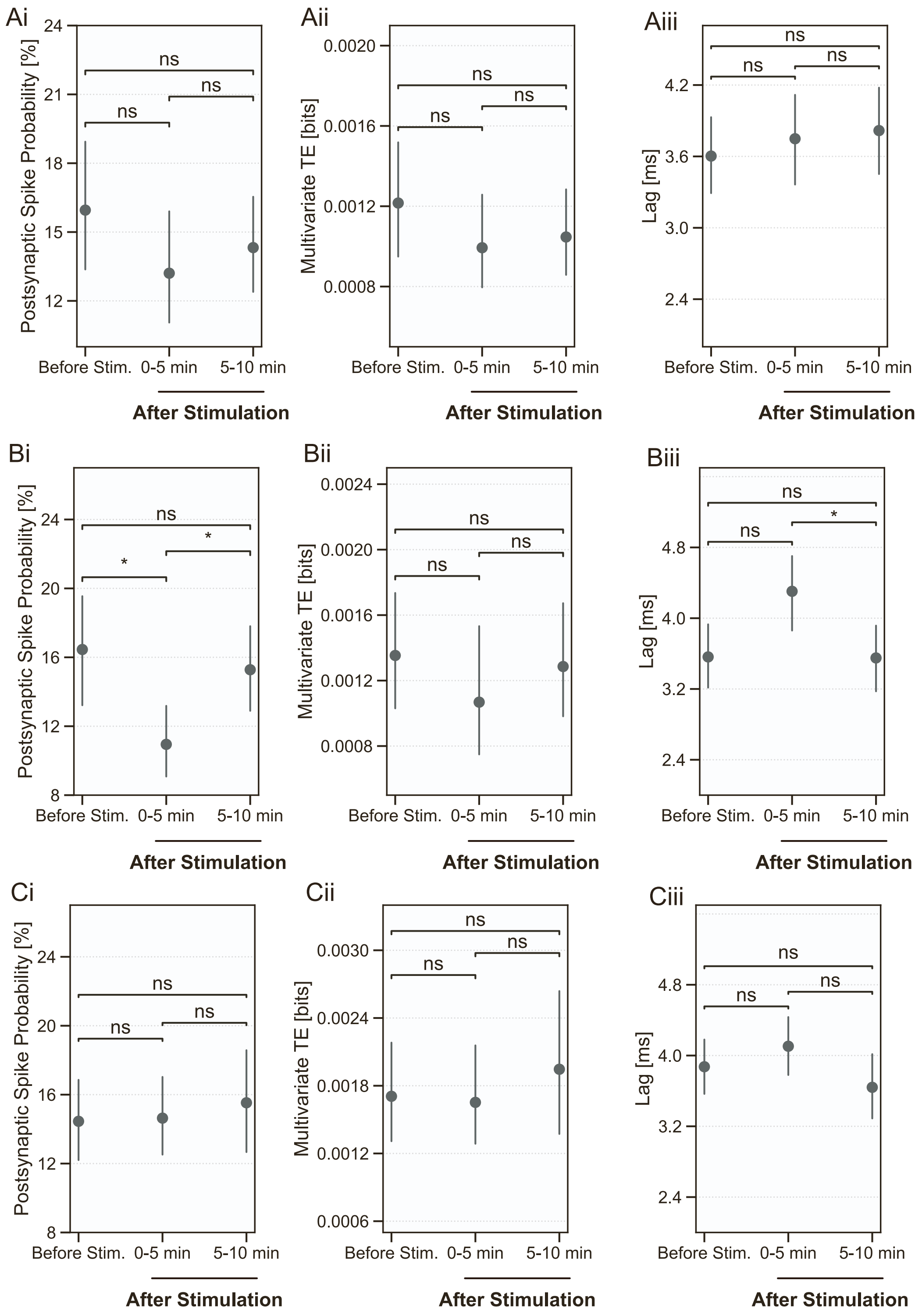


**Figure S16:** Stimulation protocol results using other stimulation frequencies. **A**) Synaptic metrics quantifying changes upon 10 Hz stimulation. Ai) Postsynaptic spike probability, Aii) multivariate transfer entropy and Aiii) the lag shows no significant changes immediately following stimulation. **B**) Synaptic metrics quantifying changes upon 20 Hz stimulation. While Bi) postsynaptic spike probability indicates synaptic weakening, Bii) multivariate transfer entropy and (iii) the lag shows no significant changes. **C**) Synaptic metrics quantifying changes upon 40 Hz stimulation. Ci) Postsynaptic spike probability, (ii) multivariate transfer entropy and Cii) the lag shows no significant changes immediately following stimulation. Data are represented as mean ± 95% confidence interval. (*$p < 0.05$, Kruskal-Wallis omnibus tests followed by Dunn's tests).

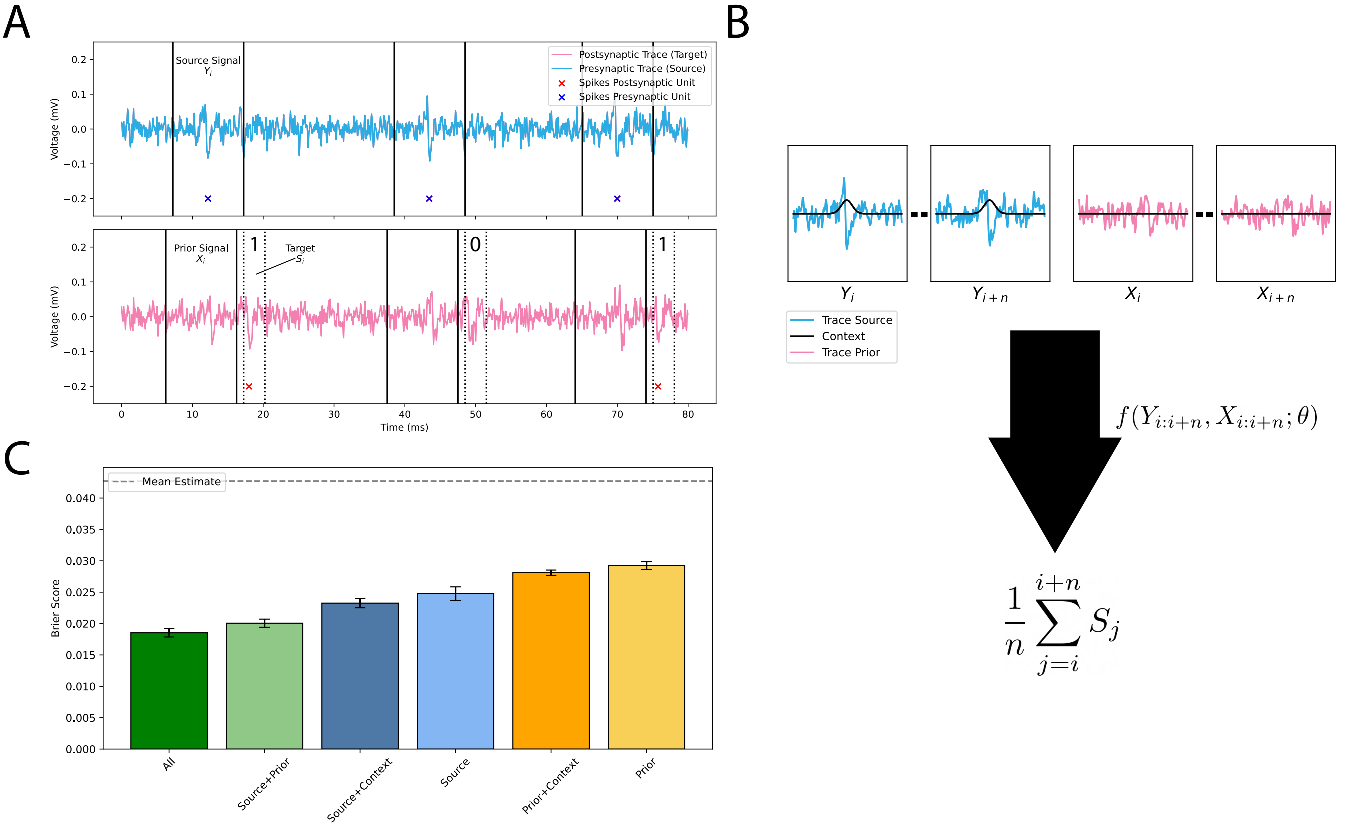


**Figure S17:** Firing probability prediction with machine learning. **A**) Illustration of sample extraction from raw extracellular signals. The target window (3 ms) is centered around the most significant lag derived from the transfer entropy between pre- and postsynaptic neuron pairs. Source and prior time windows are aligned relative to the target. The binary target is set to one if a postsynaptic spike occurs within the window, zero otherwise. **B**) Machine learning setup: a transformer model receives $n$ samples and predicts the expected firing probability. An optional context signal (spike train convolved with a Gaussian) can be added. **C**) Brier scores for various model configurations. A mean baseline, which predicts the average firing probability from the training set, is included as a reference. Bar plots show the mean ± standard deviation.

## Supplementary Tables

**Table S1:** Statistical analysis of forward directionality across PDMS microstructure types (EP, H, S). One-way ANOVA revealed a significant difference between groups. Pairwise comparisons were performed using t-tests with Bonferroni correction. Statistical significance is denoted as: ns (p $>$ 0.05), * (0.01 $<$ p $\leq$ 0.05). N=94, 245, 175 for EP, H, S respectively. Related to Figure 2.

| **One-way ANOVA:** $F = 10.20$, $p = 0.0031$ | | | | |
|---|---|---|---|---|
| **Comparison** | **t-statistic** | **p-value** | **Bonferroni-adjusted p** | **Significance** |
| EP vs. H | $-4.41$ | $0.009$ | $0.0281$ | * |
| EP vs. P | $-2.72$ | $0.03$ | $0.0897$ | ns |
| H vs. P | $0.97$ | $0.4$ | $1.000$ | ns |

**Table S2:** Statistical quantification of the synapse validation data. Kruskal-Wallis omnibus tests followed by Dunn's tests with Benjamini-Hochberg correction comparing synaptic probability and transfer entropy across different experimental timepoints: before adding the synaptic blocker, immediately after adding the blocker and one-week-after upon washout. Statistical significance is denoted as: ns ($p > 0.05$), * ($0.01 < p \leq 0.05$), ** ($0.001 < p \leq 0.01$), *** ($0.0001 < p \leq 0.001$), and **** ($p \leq 0.0001$). N=98, 27, 133 of synaptic pairs before, immediately after and one week after blocking. Related to Figure 4.

| **Metric** | **KW statistic** | **Before vs. Blocked** | **Blocked vs. Washed** | **Before vs. Washed** |
|---|---|---|---|---|
| Postsynaptic Spike Probability | P=$3.2 \cdot 10^{-24}$<br>Stat=108.2 | P=$1.1 \cdot 10^{-18}$<br>**** | P=$1.2 \cdot 10^{-20}$<br>**** | P=0.64<br>ns |
| Bivariate Transfer Entropy | P=$1.0 \cdot 10^{-25}$<br>Stat=115.0 | P=$3.1 \cdot 10^{-21}$<br>**** | P=$3.1 \cdot 10^{-21}$<br>**** | P=0.25<br>ns |

**Table S3:** Statistical quantification of the perturbation data. Kruskal-Wallis omnibus tests followed by Dunn's tests with Benjamini-Hochberg correction comparing connectivity metrics before and after stimulation. B represents baseline recording prior to stimulation, A represents the first 5 minutes post-stimulation, and $A_2$ represents the second 5 minutes post-stimulation. Statistical significance is denoted as: ns ($p > 0.05$), * ($0.01 < p \leq 0.05$), ** ($0.001 < p \leq 0.01$), *** ($0.0001 < p \leq 0.001$), and **** ($p \leq 0.0001$). Number of synaptic pairs tested N=194 across three different chips. Related to Figure 8.

| **Metric** | **KW statistic** | **B vs. A** | **A vs. $A_2$** | **B vs. $A_2$** |
|---|---|---|---|---|
| Postsynaptic Spike Probability | P=0.04<br>Stat=6.5 | P=0.04<br>* | P=0.25<br>ns | P=0.17<br>ns |
| mTE | P=0.009<br>Stat=9.5 | P=0.006<br>** | P=0.09<br>ns | P=0.15<br>ns |
| Lag | P=0.18<br>Stat=3.5 | P=0.68<br>ns | P=0.35<br>ns | P=0.21<br>ns |

**Table S4:** HH parameter analysis before and immediately after perturbation. Statistical analysis of HH model parameters comparing experimental data (number of synaptic pairs N=194 across three different chips) before and immediately after stimulation. Three different tests were applied: t-test (tests if the means of the distributions are different), KS-test (tests if the entire distributions are different), and Mann-Whitney U test (non-parametric test for distribution differences). Related to Figure 8.

| **Parameter** | **t-test** | **Sig.** | **KS-test** | **Sig.** | **MW-test** | **Sig.** |
|---|---|---|---|---|---|---|
| $d_{pre}$ | P= 0.9 | False | P=0.5 | False | P=0.8 | False |
| $d_{post}$ | P=0.02 | True | P=0.03 | True | P=0.04 | True |
| $mean_{AMPA}$ | P=0.003 | True | P=0.008 | True | P=0.001 | True |
| $std_{AMPA}$ | P=0.1 | False | P=0.03 | True | P=0.2 | False |
| $mean_{NMDA}$ | P=0.006 | True | P=0.02 | True | P=0.01 | True |
| $std_{NMDA}$ | P=0.8 | False | P=0.2 | False | P=0.3 | False |
| $1/\lambda_{pre}$ | P=0.03 | True | P=0.0006 | True | P=0.09 | False |
| $1/\lambda_{post}$ | P=0.1 | False | P=0.01 | True | P=0.09 | False |
| Syn. Loc. | P=0.03 | True | P=0.01 | True | P=0.03 | True |

**Table S5:** HH parameter analysis before and five minutes after perturbation. Statistical analysis of HH model parameters comparing experimental data (number of synaptic pairs N=194 across three different chips) before and five minutes after stimulation. Three different tests were applied: t-test (tests if the means of the distributions are different), KS-test (tests if the entire distributions are different), and Mann-Whitney test (non-parametric test for distribution differences). Related to Figure S12.

| **Parameter** | **t-test** | **Sig.** | **KS-test** | **Sig.** | **MW-test** | **Sig.** |
|---|---|---|---|---|---|---|
| $d_{pre}$ | P=0.6 | False | P=0.2 | False | P=0.8 | False |
| $d_{post}$ | P=0.08 | False | P=0.08 | False | P=0.2 | False |
| $mean_{AMPA}$ | P=0.07 | False | P=0.01 | True | P=0.007 | True |
| $std_{AMPA}$ | P=0.08 | False | P=0.5 | False | P=0.4 | False |
| $mean_{NMDA}$ | P=0.001 | True | P=0.02 | True | P=0.007 | True |
| $std_{NMDA}$ | P=0.6 | False | P=0.8 | False | P=0.9 | False |
| $1/\lambda_{pre}$ | P=0.3 | False | P=0.1 | False | P=0.4 | False |
| $1/\lambda_{post}$ | P=0.5 | False | P=0.5 | False | P=0.8 | False |
| Syn Loc. | P=0.0007 | True | P=0.0003 | True | P=0.0004 | True |

**Table S6:** High-probability vs. low-probability parameter distributions. Mann-Whitney-Wilcoxon test results comparing non-normalized absolute deviation of parameters pulled from Maximum A Posterior (MAP) and Low Probability (LP) distributions. Statistical significance is denoted as: ns ($p > 0.05$), * ($0.01 < p \leq 0.05$), ** ($0.001 < p \leq 0.01$), *** ($0.0001 < p \leq 0.001$), and **** ($p \leq 0.0001$). Related to Figure 7.

| **Metric** | **Comparison** | **P-value** | **U-statistic** |
|---|---|---|---|
| Firing Rate | Target$_{MAP}$ vs. Target$_{LP}$ | $5 \cdot 10^{-150}$ **** | $3 \cdot 10^5$ |
| Firing Rate | Source$_{MAP}$ vs. Source$_{LP}$ | $1 \cdot 10^{-58}$ **** | $5 \cdot 10^5$ |
| Conduction Speed | Source$_{MAP}$ vs. Source$_{LP}$ | $8 \cdot 10^{-94}$ **** | $4 \cdot 10^5$ |
| Conduction Speed | Target$_{MAP}$ vs. Target$_{LP}$ | $3 \cdot 10^{-159}$ **** | $3 \cdot 10^5$ |
| Synaptic Probability | MAP vs. LP | $5 \cdot 10^{-146}$ **** | $3 \cdot 10^5$ |
| Lag | MAP vs. LP | $1 \cdot 10^{-114}$ **** | $4 \cdot 10^5$ |

**Table S7:** Data preparation overview. Values used in the dataset creation for the machine learning approach.

| **Symbol** | **Value** | **Description** |
|---|---|---|
| $\Delta_{\text{source}}$ | 11 ms | Time shift applied to source window |
| $\Delta_{\text{prior}}$ | 12 ms | Time shift applied to prior window |
| $w_{\text{source}}$ | 10 ms | Duration of source window |
| $w_{\text{prior}}$ | 10 ms | Duration of prior window |
| $b$ | 3 ms | Bin width for target |
| $n$ | 10 | Number of samples concatenated as input |
